# Antiprogestins reduce epigenetic field cancerization in breast tissue of young healthy women

**DOI:** 10.1186/s13073-022-01063-5

**Published:** 2022-06-15

**Authors:** Thomas E. Bartlett, Iona Evans, Allison Jones, James E. Barrett, Shaun Haran, Daniel Reisel, Kiriaki Papaikonomou, Louise Jones, Chiara Herzog, Nora Pashayan, Bruno M. Simões, Robert B. Clarke, D. Gareth Evans, Talayeh S. Ghezelayagh, Sakthivignesh Ponandai-Srinivasan, Nageswara R. Boggavarapu, Parameswaran G. Lalitkumar, Sacha J. Howell, Rosa Ana Risques, Angelique Flöter Rådestad, Louis Dubeau, Kristina Gemzell-Danielsson, Martin Widschwendter

**Affiliations:** 1 Department of Statistical Science, University College London, London, WC1E 7HB UK; 2 Department of Women’s Cancer, UCL EGA Institute for Women’s Health, University College London, 74 Huntley Street, London, WC1E 6AU UK; 3 European Translational Oncology Prevention and Screening (EUTOPS) Institute, Universität Innsbruck, 6060 Hall in Tirol, Austria; 4 Research Institute for Biomedical Aging Research, Universität Innsbruck, 6020 Innsbruck, Austria; 5 Department of Women’s and Children’s Health, Division of Obstetrics and Gynecology, Karolinska Institutet and Karolinska University Hospital, Stockholm, Sweden; 6 Centre for Tumour Biology Department, Barts Cancer Institute, Queen Mary University of London, London, UK; 7 Department of Applied Health Research, University College London, 1-19 Torrington Place, London, WC1E 7HB UK; 8 Breast Biology Group, Manchester Breast Centre, Division of Cancer Sciences, Faculty of Biology, Medicine and Health, University of Manchester, Manchester, UK England; 9 University of Manchester, St. Mary’s Hospital, and University Hospital of South Manchester, Manchester, UK; 10 Department of Laboratory Medicine and Pathology, University of Washington, Seattle, WA 98195 USA; 11 Department of Obstetrics and Gynecology, University of Washington, Seattle, WA 98195 USA; 12 Department of Medical Oncology, The Christie NHS Foundation Trust, Manchester, UK; 13 Department of Pathology, Keck School of Medicine, USC/Norris Comprehensive Cancer Centre, University of Southern California, Los Angeles, USA

**Keywords:** Breast cancer, Prevention, Antiprogestins, *BRCA1*, Intermediate surrogate marker, Epigenetics, DNA methylation

## Abstract

**Background:**

Breast cancer is a leading cause of death in premenopausal women. Progesterone drives expansion of luminal progenitor cells, leading to the development of poor-prognostic breast cancers. However, it is not known if antagonising progesterone can prevent breast cancers in humans. We suggest that targeting progesterone signalling could be a means of reducing features which are known to promote breast cancer formation.

**Methods:**

In healthy premenopausal women with and without a BRCA mutation we studied (i) estrogen and progesterone levels in saliva over an entire menstrual cycle (*n* = 20); (ii) cancer-free normal breast-tissue from a control population who had no family or personal history of breast cancer and equivalently from *BRCA1/2* mutation carriers (*n* = 28); triple negative breast cancer (TNBC) biopsies and healthy breast tissue taken from sites surrounding the TNBC in the same individuals (*n* = 14); and biopsies of ER+ve/PR+ve stage T1–T2 cancers and healthy breast tissue taken from sites surrounding the cancer in the same individuals (*n* = 31); and (iii) DNA methylation and DNA mutations in normal breast tissue (before and after treatment) from clinical trials that assessed the potential preventative effects of vitamins and antiprogestins (mifepristone and ulipristal acetate; *n* = 44).

**Results:**

Daily levels of progesterone were higher throughout the menstrual cycle of *BRCA1/2* mutation carriers, raising the prospect of targeting progesterone signalling as a means of cancer risk reduction in this population. Furthermore, breast field cancerization DNA methylation signatures reflective of (i) the mitotic age of normal breast epithelium and (ii) the proportion of luminal progenitor cells were increased in breast cancers, indicating that luminal progenitor cells with elevated replicative age are more prone to malignant transformation. The progesterone receptor antagonist mifepristone reduced both the mitotic age and the proportion of luminal progenitor cells in normal breast tissue of all control women and in 64% of *BRCA1/2* mutation carriers. These findings were validated by an alternate progesterone receptor antagonist, ulipristal acetate, which yielded similar results. Importantly, mifepristone reduced both the *TP53* mutation frequency as well as the number of *TP53* mutations in mitotic-age-responders.

**Conclusions:**

These data support the potential usage of antiprogestins for primary prevention of poor-prognostic breast cancers.

**Trial registration:**

Clinical trial 1 *Mifepristone treatment prior to insertion of a levonorgestrel releasing intrauterine system for improved bleeding control – a randomized controlled trial*, clinicaltrialsregister.eu, 2009-009014-40; registered on 20 July 2009.

Clinical trial 2 *The effect of a progesterone receptor modulator on breast tissue in women with BRCA1 and 2 mutations*, clinicaltrials.gov, NCT01898312; registered on 07 May 2013.

Clinical trial 3 *A pilot prevention study of the effects of the anti- progestin Ulipristal Acetate (UA) on surrogate markers of breast cancer risk*, clinicaltrialsregister.eu, 2015-001587-19; registered on 15 July 2015.

**Supplementary Information:**

The online version contains supplementary material available at 10.1186/s13073-022-01063-5.

## Background

Metastatic cancers are rarely curable regardless of their organ of origin. Primary prevention is particularly important for diseases such as breast cancer that have a tendency to disseminate very early during tumour formation [1, 2]. Adoption of treatment strategies based on established targets and disease surveillance using surrogate markers, akin to those applied in the setting of cardiovascular disease, have been advocated for cancer [3, 4] and particularly for highly prevalent cancers, such as breast cancer [5]. In a recent international consensus conference, the identification of biomarkers representative of field defects (i.e. microscopic normal tissue predisposed to cancer formation) in normal breast tissues that are associated with breast cancer predisposition was highlighted as an unmet need [6]. In particular, the identification of intermediate surrogate biomarkers (i.e. substitutes for clinical endpoints) for monitoring response to preventive measures in normal tissues was singled out as the highest priority.

Women with a *BRCA1* mutation have a >65% lifetime risk of triple negative breast carcinoma [7–9], a subtype which accounts for 10–15% of breast cancers [10] and which are thought to originate in luminal progenitor cells (reviewed in [11]) and have a particularly poor prognosis [12] and a high frequency of *TP53* mutations [13]. The idea that germline mutations in the ubiquitously expressed *BRCA1* gene drive cancer development primarily via a classical tumour-suppressor mechanism triggered by a reduced “chromosome custodian” function [14] does not account for the organ-specificity of the cancers associated with this carrier state [15]. We previously reported evidence for cell-nonautonomous mechanisms of cancer predisposition in humans and experimental animals carrying germline *BRCA1* or *Brca1* mutations [16–21]. By definition, such mechanisms are driven by consequences of this carrier state on organs that are different than (and are upstream of) those that are cancer-prone. For example, *Brca1-*deficient mice and human *BRCA1* mutation carriers show elevated levels of ovarian-derived sex steroid hormones [19, 20]. This results in progesterone, the predominant sex steroid hormone during the post-ovulatory phase of the mouse estrous or human menstrual cycle, triggering increased levels of RANKL in the breast tissue [22–26] and reduced breast tissue and circulating levels of the physiological RANKL-antagonist Osteoprotegerin [22]. The ensuing expansion of hormone receptor negative mammary stem cells leads to malignant transformation in mice [27]. Progesterone increases RANKL expression in progesterone receptor positive mature luminal epithelial cells, which acts in a paracrine manner on RANK+ luminal progenitor cells in the breast epithelium [11]. Furthermore, recent evidence from randomised clinical trials established a role of progestins in triggering aggressive forms of breast cancer [28].

These cell non-autonomous consequences of *BRCA* germline mutations potentially could be prevented by the administration of progesterone receptor antagonists such as mifepristone in women with a high lifetime genetic risk for these cancers. Indeed, this agent has prevented malignant formation in *Brca1*-deficient mice [29].

Here we sought to investigate the merit of targeting progesterone signalling as a means of cancer prevention by (i) comparing the levels of progesterone during one menstrual cycle in women with and without a *BRCA* mutation, (ii) developing markers which are indicative of a progesterone-mediated field cancerization in the breast and (iii) examining the ability of progesterone receptor antagonists (mifepristone and ulipristal acetate) to reduce the level of field cancerization in normal breast tissue.

## Methods

### Hormonal saliva data-set

*BRCA1/2* mutation carriers and confirmed non-carriers controls were recruited as part of the BRCA Unite Research Study (REC reference: 14/LO/1633; IRAS ID: 53431). The enrolment criteria were (1) Premenopausal women, 18 to 45 years of age; (2) with good general health; (3) regular menstrual cycles (25-35 days); (4) not taking any concurrent hormonal medication or in the past three months; (5) not pregnant or breastfeeding; (6) no previous diagnosis of cancer; (7) no previous risk-reducing surgical removal of both ovaries and Fallopian Tubes; (8) willing and able to participate after giving informed consent; and (9) women with confirmatory testing of *BRCA1/2* mutation. Participants were asked to collect a daily saliva sample each morning (~1 ml), over the course of one full menstrual cycle (the cycle length was defined as the time between the first day of vaginal bleeding in subsequent menstrual cycles). Salivary hormone levels were measured using enzyme immunoassay kits from Salimetrics; for Progesterone (#1-1502-5) and Estradiol (#1-4702-5), following the manufacturer’s instructions. Visual aids and additional written instructions were made available. Collected saliva samples were immediately frozen in a domestic freezer at ~ −20^o^C. Once the collection was complete over one menstrual cycle, frozen samples were transferred to the laboratory using icepacks and a thermally insulated bag before being transferred into −80^o^C. Participants completed a sample log to record cycle length and any issues with the samples collected from individuals.

### DNA methylation data sets

**Fig. 1:**
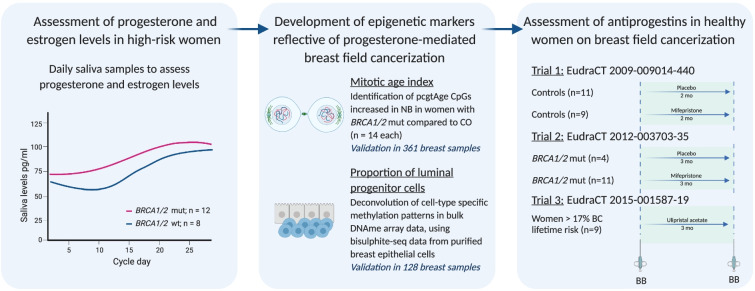
Summary of the rationale and design of the study. Abbreviations: mut (mutation); wt (wild-type); pcgt (polycomb-group targets); NB (normal breast); CO (control); BB (breast biopsy); BC (breast cancer)

Four sets of samples were used in the DNAme microarray analyses:

*DNAme Set 1:* to establish and validate the two components of the breast field cancerization (Fig. 1). Cancer-free normal breast-tissue samples from a control population who had no family or personal history of breast cancer (*n =* 14, average age at cosmetic surgery 31 years) and from *BRCA1/2* mutation carriers (*n =* 14, average age at surgery 36 years); triple negative breast cancer (TNBC) biopsies and healthy breast tissue samples taken from sites surrounding the TNBC in the same individuals (*n =* 14, average age at surgery 43 years).

*DNAme Set 2:* to assess the tests in ER+ve/PR+ve cancers. Biopsies from ER+ve/PR+ve stage T1–T2 cancers and healthy breast tissue samples taken from sites surrounding the cancer in the same individuals (*n* = 31, average age at surgery 51 years, including 6 node-positive, 5 grade 3, and 4 HER2+).

Samples used for DNAme Set 1 and DNAme Set 2 were obtained from https://breastcancernow.org/breast-cancer-research/breast-cancer-now-tissue-bank

*DNAme Set 3 (Clinical Trials 1 and 2):* to assess the performance of the *WID-Breast29* test monitoring mifepristone-preventive measures in real time. The samples were obtained from two Clinical Trials (Fig.1; Additional file 1: Fig.S3-4) as follows.

Clinical Trial 1 was “*Mifepristone treatment prior to insertion of a levonorgestrel releasing intrauterine system for improved bleeding control – a randomized controlled trial*” [30] (EudraCT number: 2009-009014-40; Registration date: 20/07/2009; Regional ethical review board at Karolinska Institutet permit 2009/144-31/4) (Additional file 2). This study conformed to the principles of the Helsinki Declaration. The trial was prospectively registered, and the date of first patient enrolment was 24 November 2009; the last subject was screened in November 2013 and the last study contact was in January 2015. Data were collected at the Karolinska University Hospital, Stockholm, Sweden. Mifepristone was obtained from Exelgyn, Paris, France, and visually indistinguishable vitamin B from Recip, Stockholm, Sweden. The tablets were divided into four parts: one quarter of the comparator or the mifepristone 200mg tablet was taken orally every other day. The enrolment criteria were (1) women 18 years of age or above eligible for LNG-IUS; (2) regular and normal menstrual cycles lasting 25–35 days; (3) willing and able to participate after the study has been explained; and (4) signed informed consent was given. The exclusion criteria were (1) allergy or contraindication to mifepristone or LNG-IUS, (2) any hormonal treatment used within 2 months prior to study start, and (3) a medical condition or disease that requires special treatment, care or precaution (e.g. corticosteroid or anticoagulant therapy). The primary objective was to assess the effect of mifepristone pre-treatment on the initial bleeding pattern after insertion of a levonorgestrel-releasing intrauterine system. Assessment was made using vaginal ultrasound scan at the beginning of the study and an ultrasound scan performed prior to the endometrial biopsies. The secondary objectives were to (1) study the effect of mifepristone on the breast tissue and (2) study the effects of mifepristone and LNG on the endometrium. This assessment was made by a core needle breast biopsy collected at baseline and following two months of pretreatment prior to IUS placement, and endometrial biopsy which was obtained at baseline prior to start of pre-treatment and at three months following placement of the LNG-IUS 52mg.

Clinical Trial 2 was “*The effect of a progesterone receptor modulator on breast tissue in women with BRCA1 and 2 mutations*” (EudraCT registration number: 2012-003703-35; registration date: 07/05/2013; regional ethical review board at Karolinska Institutet permit 2012/729 31/1) (Additional file 3). This study conformed to the principles of the Helsinki Declaration. This trial was prospectively registered, the date of the first patient enrolment was 2 April 2015, and the date of the last patient enrolment was 14 October 2021. Data were collected at WHO-centre, Dept. of Women’s and Children’s Health, Division of Obstetrics and Gynecology Karolinska Institutet and Karolinska University Hospital, Stockholm, Sweden. Mifepristone was obtained from Exelgyn, Paris, France, and visually indistinguishable vitamin B from Recip, Stockholm, Sweden. The tablets were divided into four parts: one quarter of the comparator or the mifepristone 200mg tablet was taken orally every other day. The enrolment criteria were (1) Premenopausal women, ≥ 18 years of age; (2) with good general health; (3) regular menstrual cycles (25–35 days); (4) willing and able to participate after giving informed consent; and (5) women having *BRCA1/2* mutation and have decided to undergo risk-reducing mastectomy. The exclusion criteria were (1) any hormonal treatment used within 2 months prior to study start and (2) any contraindication to mifepristone. The exclusion criteria were amended to include previous/current diagnosis of hepatitis -diagnosis of Cirrhosis of the liver. The primary outcome of this study was to assess the safety and effect of treatment with mifepristone on epithelial cell proliferation in human breast tissue in women with *BRCA1* or *2* mutations prior to protective mastectomy. The epithelial cell content was assessed by DNA methylation microarray. The primary endpoint was hence defined as a 20% reduction in breast cell proliferation at 12 weeks treatment. The Secondary outcomes were (1) vital signs and safety laboratory analysis; (2) side effects and adverse events; (3) endometrial effects; (4) ovarian effects; (5) acceptability; (6) breast symptom evaluation; and (7) expression of oestrogen receptor (ER), progesterone receptor (PR) and androgen receptor (AR), as well as apoptosis, proliferation and biomarkers for the development of cancer in the breast tissue and collagen content, before and at the end of three months mifepristone or vitamin treatment. Assessment of secondary outcomes is by differential expression analysis of genes specifically in the pathways involving apoptotic and cell proliferation by microarray, and significantly altered factors observed in the microarray analysis, including PTEN, Bcl-2 and Ki-67 along with steroid receptors (ERα, ERβ, ERβcx, PR-A, PR-B, AR), as well as collagen content, will be reconfirmed by real-time PCR and the protein expression studied by immunohistochemistry using breast tissues collected at the beginning and end of treatment. Secondary endpoints are (1) type of side effects and adverse events and (2) endometrial morphology and rate of PAEC (progesterone receptor modulator associated endometrial changes).

From Clinical Trial 1, 12 women from each of the vitamin and mifepristone groups were included in this study; of these, 11 and 9 women, respectively, had provided sufficient DNA from the pre- and post-treatment biopsies for subsequent processing and analysis. In addition, 15 women from the mifepristone group were also used to extract RNA and subsequent downstream processing and analysis were performed to check RANKL expression and cell type proportion analysis. In Trial 2, 7/8 and 14/16 women in the vitamin and mifepristone groups, respectively, had provided sufficient pre-treatment biopsies, of which 4 and 11, respectively, later also provided a post-treatment sample with sufficient DNA yield (Additional file 1: Fig. S3-4). The age range for women who had DNAme profiles in the pre- and post-treatment sample and were treated with vitamins ranged from 20 to 51 years and women who were treated with mifepristone ranged from 21 to 39 years.

*DNAme Set 4 (Clinical Trial 3):* to assess the performance of the breast field cancerization tests monitoring ulipristal acetate preventive measures in real time. Clinical Trial 3 was “*A pilot prevention study of the effects of the anti- progestin Ulipristal Acetate (UA) on surrogate markers of breast cancer risk*” (EudraCT registration number: 2015-001587-19; registration date: 15/07/2015; Greater Manchester – South, Research Ethics Committee number 15/NW/0478) (Additional file 4). This study conformed to the principles of the Helsinki Declaration. This trial was prospectively registered, the first patient was consented in March 2016 and the last in March 2019. The main data collection took place at Manchester University NHS Foundation Trust, Manchester, U.K. Commercially available ulipristal acetate 5 mg tablets were administered.

The enrolment criteria were (1) premenopausal females aged between 25 and 45 years; (2) regular menses defined as date of onset of last menstrual period +/− 3 days of expected; (3) willing to use an acceptable method of contraception from screening to 2 weeks after study drug discontinuation (see the “Restrictions” section below); (4) increased risk of breast cancer (≥1:6 increased lifetime risk assessed by Tyrer-Cuzick v8); (5) ovulatory menstrual cycles defined as serum progesterone ≥15nmol in the luteal phase of the menstrual cycle (in the 2 weeks prior to expected onset of menses); and (6) willing and able to provide informed consent to undergo all trial procedures. The exclusion criteria were (1) personal history of breast, uterine, cervical or ovarian cancer; (2) breast feeding within the last 3 months; (3) pregnant or planning for pregnancy in the next 6 months. Pregnancy must be excluded with serum βhCG; (4) known hypersensitivity to radiological contrast media; (5) known hypersensitivity to ulipristal acetate or any of its excipients (microcrystalline cellulose, mannitol, croscarmellose sodium, talc, magnesium stearate); (6) current treatment with anti-estrogens (e.g. tamoxifen or raloxifene), GnRH analogue therapy (e.g. goserelin or buserelin) or hormonal contraceptives including androgens such as cyproterone acetate. Such treatments must have been stopped for at least 6 months and regular menstrual cycles resumed; oral corticosteroids at any dose, these must have been stopped for at least 1 month with low likelihood that retreatment will be required; antiplatelet or anticoagulant therapy; (7) moderate or potent inhibitors of CYP3A4, potent inducers of CYP3A4, APTT and PT above the upper limit of the normal institutional ranges; (8) previous/current diagnosis of hepatitis; (9) diagnosis of cirrhosis of the liver; (10) co-morbidity that would put the patient at increased risk such as recognised bleeding diathesis, moderate to severe hepatic impairment, moderate or severe renal impairment, severe asthma not adequately controlled with corticosteroids; (11) prior breast enhancement/augmentation surgery; and (12) genital bleeding of unknown aetiology or for reasons other than uterine fibroids. The primary endpoint for Clinical Trial 3 was the change in epithelial cell proliferation measured by %Ki67 IHC staining with 3 months’ treatment with ulipristal acetate. The secondary endpoints were (1) percentage of luminal, basal and mixed colonies by morphological analysis of adherent feeder layer assay; (2) percentage of luminal progenitor cells (EPCAM+/CD49f+) by FACS analysis; (3) tissue stiffness assessed as the reduced indentation modulus by atomic force microscopy; (4) mean tissue section percentage fibrillar collagen assessed by picrosirius red staining and polarised light microscopy; (5) background parenchymal enhancement assessed by magnetic resonance imaging (MRI); (6) the side effect profile of UA in this patient population assessed by CTCAE v4.03; (7) the relative change in Ki67 with UA treatment between those with and without known mutation in *BRCA1/2* genes. Exploratory analyses were (1) the changes in expression of individual genes and key pathways induced by UA therapy; (2) the changes in key stem cell and PgR target proteins induced by UA therapy; (3) the changes in fibroglandular volume and other MRI/US elastography biomarkers; (4) the changes in fibroglandular and fat tissue stiffness assessed by ultrasound elastography and acoustic radiation force imaging (ARFI); and (5) the changes in breast impedance measured at baseline and 3 months. Additional physical harms are noted as originating from blood tests that would otherwise not be performed.

After 12 weeks of ulipristal acetate 5mg daily, MRI and vacuum-assisted breast biopsy (VAB) of the contralateral breast were performed. Only women < 38 years of age (*n* = 9) were included in the subsequent DNAme analysis (DNAme Set 4), so that the age range would more closely match that of DNAme Set 3 (Clinical Trials 1–2).

### DNA methylation analysis

DNA samples were normalised to 25 ng/μl. We bisulfite-modified 500 ng of tissue DNA using the DNA methylation Lightning Mag Prep Kit from Zymo Research (cat number D5047) on a Hamilton Star liquid handling platform. We eluted 15 μl of the bisulfite-converted DNA, which was then subjected to methylation analysis on the Illumina InfiniumMethylation EPIC BeadChip (Illumina, CA, USA) at UCL Genomics, according to the manufacturer’s standard protocol. Details on DNA methylation data analysis are given in Additional file 1.

### RNA extraction, cDNA synthesis, and real-time PCR

RNA extraction from paired pre- and post-mifepristone treated healthy breast tissues was performed using Trizol reagent with Purelink RNA mini kit (Life Technologies, USA) as per the manufacturer’s protocol and subsequently converted to complementary DNAs (cDNAs) using SuperScript® VILOTM kit (Invitrogen®, Thermo Fisher Scientific, Waltham, USA). 10 ng of pre-diluted complementary DNA were used in triplicates in real-time polymerase chain reaction (RT-PCR) with TaqMan® fast advanced master mix (cat no. 4444554) and TaqMan® gene expression probe/primer for RANKL (Cat no. Hs00243522_m1) and analysed using StepONE RT-PCR (Applied Biosystems, USA). Ribosomal RNA 18s (Cat no. 4319413E) was used as a housekeeping gene to normalise the gene expression of RANKL and the relative expression fold change was calculated for the above-paired treatment groups using standard formula 2^-ΔΔCT^.

### RNA sequencing and analysis

DNA libraries for next-generation sequencing were constructed from RNA of paired pre- and post-mifepristone treated healthy breast tissues using the well-established Smart-seq2 protocol [31]. The tagmentation step was performed using the Nextera XT kit (Illumina) and sequencing was performed using Illumina NextSeq 550®. The transcriptomic data were deposited in NCBI’s Gene Expression Omnibus (GEO). The sequencer reads were analysed using pre-designed modules available for RNA sequencing using Partek Flow Genomic Analysis Software (Partek, St. Louis, Missouri, USA). Briefly, sequenced raw FASTQ files were trimmed for adapters and contaminants such as ribosomal DNA and mitochondrial DNA. Post-quality control reads were mapped to the reference genome hg38 using STAR aligner with default settings, further filtered and quantified to coding transcripts/genes using hg38 assembly and Ensemble transcripts release 91. Gene counts were obtained after filtering for regions fully or partially spanned within exon regions.

### *TP53* mutational analysis by Duplex Sequencing

For a subset of 8 women from Clinical Trials 1 and 2 (*‘Mifepristone treatment prior to insertion of a levonorgestrel releasing intrauterine system for improved bleeding control – a randomized controlled trial’* and ‘*The effect of a progesterone receptor modulator on breast tissue in women with BRCA1 and 2 mutations’*) [i.e. women who showed a decrease of the *Wid-Breast29* index after mifepristone (responders; *n* = 5) and who did not show a decrease (non-responders, *n* = 3)], *TP53* mutations were analysed using Duplex Sequencing [32, 33] in DNA extracted from normal breast tissue collected before and after mifepristone treatment. Two hundred nanograms of DNA was sonicated using a Covaris system and processed with Duplex Sequencing kits according to the manufacturer’s instructions (TwinStrand Biosciences, Seattle, WA, USA). Briefly, sheared DNA was end-repaired, A-tailed, ligated to Duplex adapters (which contain double-stranded molecular barcodes), and PCR amplified. Then the *TP53* coding region was captured by hybridization with biotinylated probes in two successive rounds to ensure sufficient target enrichment, as previously described [34]. Indexed libraries were pooled and sequenced in an Illumina MiSeq using v2 300 cycle kits. Data analysis was performed using the standard Duplex Sequencing pipeline [32] with updated modifications (https://github.com/Kennedy-Lab-UW/Duplex-Seq-Pipeline). For each sample, we sequenced an average of 5.2M duplex nucleotides in coding *TP53* exons, corresponding to an average of 3879x duplex depth. For each sample, we calculated *TP53* mutation frequency as the number of identified mutant positions divided by the total number of nucleotides sequenced in the coding region. SNPs and intronic mutations, except for splice sites, were excluded from mutation analysis. *TP53* hotspot mutations were defined as the top 1% most frequent mutations in breast cancer according to the COSMIC database.

### Matched gene expression and DNAme analysis

Gene expression levels were measured by microarray on 216 breast cancer invasive carcinoma samples and 38 healthy control breast tissue samples, and DNAme levels were also measured by microarray on the same 216 + 38 samples. These publicly available data were downloaded from the TCGA repository (The Cancer Genome Atlas [35]). For further details please see Additional file 1.

### Tumour gene expression microarray DNAme analysis

DNAme data for 257 breast cancer invasive carcinoma samples with associated clinical data were downloaded from the TCGA repository (The Cancer Genome Atlas [35]). For further details please see Additional file 1.

### Statistical analysis

The *WID-Breast29* index was defined by selecting a subset of the 385 CpG loci contributing to the *pcgtAge* index [36] (a measure of ‘mitotic age’), which showed in *DNAme Set 1* increased methylation levels in the normal breast tissue of *BRCA1/2* mutation carriers compared to controls (details in Additional file 1) resulting in 37 CpGS (Additional file 1: Table S2): the *WID-Breast29* index is defined as the mean methylation level of these 37 CpGs in a sample.

DNAme is well established as a basis for estimating tissue cell-type composition in terms of the general cell subtypes epithelial, fibroblast, fat, and immune. We also developed a new algorithm [37], to estimate the proportions of the breast epithelial compartment in terms of basal, luminal progenitor, and mature luminal cells. We have made this software tool freely available as an R package from: https://github.com/tombartlett/BreastEpithelialSubtypes

## Results

Our intention was to develop a new strategy to prevent poor-prognostic breast cancers in high-risk women. Hence, we (i) assessed daily progesterone and oestrogen levels over one entire menstrual cycle in women with and without a *BRCA* mutation (*Hormonal Saliva Data-Set*), (ii) developed two epigenetic markers which are able to quantify the progesterone-driven breast field cancerization/defects (i.e. signatures to quantify cells with high mitotic age and to assess the proportion of luminal progenitors cells as the cells of origin for triple negative breast cancers, *DNAme Set 1*) and finally (iii) assess whether progesterone antagonists are able to reduce this breast cancerization field defect in as-yet unaffected young women (DNAme Sets 3–4 from Clinical Trials 1–3: *‘Mifepristone treatment prior to insertion of a levonorgestrel releasing intrauterine system for improved bleeding control – a randomized controlled trial’*, *‘The effect of a progesterone receptor modulator on breast tissue in women with BRCA1 and 2 mutations’* and *‘A pilot prevention study of the effects of the anti- progestin Ulipristal Acetate (UA) on surrogate markers of breast cancer risk’*) (Fig. 1).

### Association of the *BRCA* mutation carrier state on sex steroid hormone levels throughout the entire menstrual cycle

**Fig. 2:**
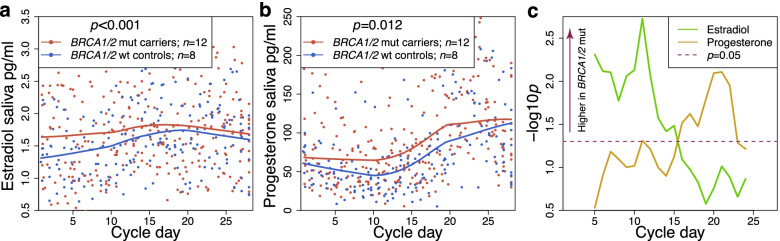
Effect of the *BRCA1/2* mutation carrier states on estradiol and progesterone levels during menstrual cycle progression. Estradiol and progesterone hormone levels were significantly greater over one cycle in *BRCA1/2* mutation carriers (*n* = 12) than in controls (*n* = 8): **a** and **b** show the time-series (respectively) for estradiol and progesterone (normalised by cycle length) with one-week moving average lines. **c** One-week moving windows (as used to plot the moving average lines in **a** and **b**) were used to assess how the relative increase in hormone levels in *BRCA1/2* mutation carriers (compared to controls) varies during the cycle. All significances were assessed with *t*-tests

We first compared daily estradiol and progesterone levels (*Hormonal Saliva Data-Set*) in saliva samples (which is a good proxy for serum levels [38]) spanning one entire menstrual cycle in *BRCA1* (*n* = 9) and *BRCA2* (*n* = 3) germline mutation carriers (cases) and confirmed non-carriers (wild type controls, *n* = 8), (Additional file 1: Table S1a). Both estradiol (Fig. 2a) and progesterone (Fig. 2b) were higher in mutation carriers compared to controls. The effects seen in levels of estradiol, which were consistent with those reported earlier in a mouse model [19], were limited to the oestrogen-dominant pre-ovulatory (follicular) phase. The effects for progesterone were seen during the entire cycle but resulted in further, statistically significant, increases in the levels of this hormone during the progesterone-dominant post-ovulatory (luteal) phase (Fig. 2c). When restricting the analysis solely to *BRCA1* or to *BRCA2* carriers (Additional file 1: Fig. S1) it is of note that estradiol was higher in both *BRCA1* and *BRCA2* mutation carriers whereas the luteal progesterone increase was only visible in *BRCA1* carriers—i.e. the group of *BRCA* carriers who are at risk for developing the highly aggressive triple negative breast cancer originating from luminal progenitor cells [11]. We propose that high estradiol levels during the pre-ovulatory phase lead to progesterone receptor upregulation in the breast (and likely other hormone-dependent tissues including the endometrium) that acts synergistically with higher progesterone levels in order to enhance progesterone signalling during the luteal phase [39].

### A DNA methylation signature surrogate for replicative age associated with cancer risk

**Fig. 3:**
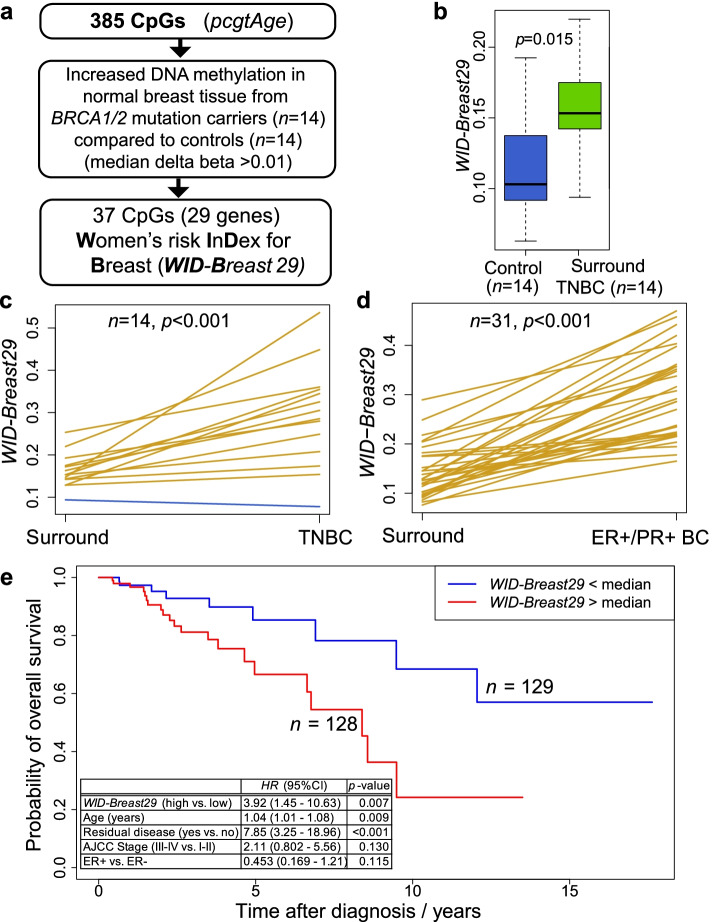
*WID-Breast29* score in triple negative breast cancer (TNBC) and adjacent normal tissues. **a** The *WID-Breast29* score is based on 37 CpGs from 29 genes and **b** was significantly greater in normal breast tissue surrounding TNBC (*n* = 14) when compared to normal tissue from cancer-free women (*n* = 14); significance was assessed with the *t*-test. **c** The *WID-Breast29* score also increased significantly when transitioning from normal surrounding tissue to TNBC (*n* = 14) or **d** ER+ve/PR+ve breast cancer (*n* = 31); gold lines indicate increasing and blue decreasing values from surround normal to the cancer tissue in individual breast cancer patients; significance was assessed with the paired-sample *t*-test. **e** The *WID-Breast29* score was also significantly associated with patient survival outcome in 257 TCGA breast cancer samples (after adjusting for covariates); significance was assessed by *z*-tests on the Wald statistics after fitting a multivariate Cox proportional hazards model

The mitotic age of normal stem cells is strongly associated with cancer risk [40, 41]. Increased progesterone signalling during the post-ovulatory phase leads to increased proliferation and thus accelerated mitotic ageing in the mammary epithelium stem cell pool. A biomarker with a fixed and stable signal such as a DNA-based marker would be strongly preferred to evaluate replicative age. We previously reported that DNAme of polycomb-group target genes is highly prevalent in cancer [42] and also increases with chronological age [43]. We subsequently identified potential DNA methylation sites (CpG dinucleotides) within polycomb-group target genes that define a tick rate that correlates with the estimated number of lifetime accumulated stem cell divisions (mitotic age) in normal tissues [36]. Here we further refined this mitotic clock, called *pcgtAge,* to generate a breast tissue-specific epigenetic index that includes a subset of 37 CpGs in 29 genes (Additional file 1: Table S2) selected from the 385 CpGs in the original *pcgtAge* panel [36]. These CpGs were selected based on methylation scores with median methylation level at least 0.01 greater in histologically normal breast tissue of *BRCA1/2* mutation carriers (*n* = 14) compared to cancer-free women with no family or personal history of breast cancer (*n* = 14), (Fig. 3a) (*DNAme Set 1*, Materials and Methods). As the *pcgtAge* CpGs were originally defined as CpGs in regulatory regions of polycomb-group targets, these CpGs are all in gene-promoters (within 200bp upstream of the transcription start site). As expected of CpGs in regulatory regions, all 37 are in CpG island (CGI) or associated regions (24 in CGIs and 13 in CGI ‘shore’ or ‘shelf’ regions). This index, named “*WID-Breast29*” (Women’s cancer risk IDentification for Breast 29), scored higher in normal breast tissues surrounding triple negative breast carcinoma (*n* = 14) than in normal breast tissue from women with no evidence of cancer (*n* = 14) (Fig. 3b) (*DNAme Set 1*). Importantly, the *WID-Breast29* index was consistently higher in triple negative breast carcinomas compared to histologically normal tissues surrounding the cancer in 13 of 14 women (Fig. 3c; Additional file 1: Table S3A) independent of variations in epithelial cell density [44] (Additional file 1: Fig. S2). We conclude that the mitotic age of normal mammary epithelium in the vicinity of triple negative breast carcinoma is higher than in cancer free breast tissue, whilst the highest level of the index was recorded in breast cancer cells. The *WID-Breast29* index was also significantly higher in 31 oestrogen receptor (ER) and progesterone receptor (PR) positive breast cancers (*DNAme Set 2*, Additional file 1: Table S3B) compared to the adjacent normal breast tissue (Fig. 3d). The potential of the *WID-Breast29* index as a clinical biomarker is underscored by a 3.6-fold (95% CI 1.31-9.64; *p*=0.013) increased risk of death per unit time in the “high *WID-Breast29*” group in 257 TCGA invasive breast cancer samples (*TCGA Breast Tumour Reference Set*, Fig. 3e) after adjusting for chronological age, disease stage, and residual disease status. We included all available TCGA samples with matched DNAme and patient survival data, because our hypothesis was that the *WID-Breast29* index is a measure of breast specific replicative age. Thus, any cancer (irrespective of histological differentiation or hormone status) with a high replicative age is expected to do worse compared to a cancer with a low replicative age.

### Evaluation of the proportion of different epithelial subtypes using a DNA methylation signature

**Fig. 4:**
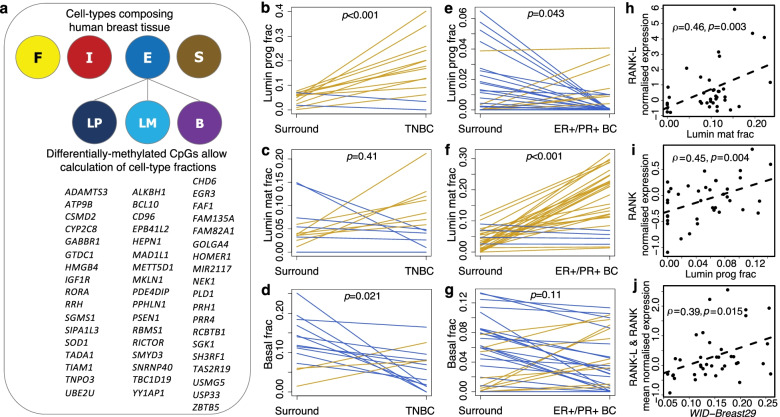
Assessment of breast epithelium composition in TNBC. **a** Fractional composition of breast-tissue samples was assessed using a well-validated algorithm [37], based on our custom-designed DNAme reference profiles for breast epithelial cell subtypes (Materials and Methods). **b** There was a highly significant increase in luminal progenitor cell concentration in TNBC (compared to normal surrounding tissue in the same volunteers, *n* = 14), whereas **c** the mature luminal cell proportion was unchanged and the **d** basal cell fraction decreased. **e** Comparing normal tissues in the same volunteers (*n* = 31) to ER+ve/PR+ve breast cancers, there was a decrease in luminal progenitors and **f** a highly significant increase in mature luminal cells and **g** no changes were noted in basal cells; gold lines indicate increasing and blue decreasing values from surround normal to the cancer tissue in individual breast cancer patients (**b-g**). **h** RANKL and **i** RANK expression (respectively) were significantly correlated with mature luminal and luminal progenitor cell proportion in 38 healthy breast tissue samples from TCGA; **j** the mean of the normalised RANKL and RANK expression levels was significantly correlated with the *WID-Breast29* index in the same 38 samples. Significances in **b–g** were assessed with the paired-sample *t*-test, and those in **h–j** were assessed with Pearson’s correlation test. F, Fat cells; I, Immune cells; E, Epithelial cells; S, Stromal cells; LP, Luminal Progenitors; LM, Luminal Mature; B, Basal

The data presented thus far suggest not only that the replicative age of the mammary epithelium is accelerated in *BRCA* mutation carriers, but also that it is further accelerated in cells adjacent to cancer. This supports the notion that a high *WID-Breast29* index is one component defining a breast field cancerization defect. Progesterone triggers RANKL secretion in mature progesterone receptor-positive luminal cells, specifically leading to the expansion of hormone receptor-negative luminal progenitor cells [11]. However, the possibility remains that our findings of increased average replicative age in breast tissues adjacent to cancer cells are merely a reflection of differences in cellular composition. We therefore examined the cellular composition of all breast tissue samples by extending a well-validated tool for cell-type deconvolution [44]. We did so by applying this algorithm sequentially, first estimating the proportions of four main cell types (epithelial, adipose, stromal, and haematopoietic), and then further decomposing the epithelial compartment into luminal progenitor, mature luminal, and basal subtypes [37] (Fig. 4a), for which we assembled our own custom-defined DNA methylation reference profiles for these epithelial subtypes based on previously published whole-genome bisulphite-sequencing data [45] (Additional file 1: Table S4; Methods). A marked increase in the number of luminal progenitor cells was noted in triple negative breast cancer compared to adjacent histologically normal breast tissue from the same respective patients (Fig. 4b). This is in alignment with the proposition that luminal progenitor cells are the cells of origin for triple negative breast cancers. The number of mature luminal cells remained unchanged (Fig. 4c) whilst that of basal cells decreased (Fig. 4d). This is in contrast with ER/PR positive breast cancers, where luminal progenitors were observed to proportionally decrease (Fig. 4e) whilst the number of mature luminal cells showed a marked increase (Fig. 4f)—consistent with the view that mature luminal cells are the cells of origin for ER/PR positive breast cancers—whilst basal cell proportions remained unchanged (Fig. 4g). Publicly available data from 38 healthy breast tissue samples (the *TCGA Matched Gene Expression and DNAme Set*) [35] showed that RANKL and RANK expression were significantly correlated with the proportion of mature luminal and luminal progenitor cells, respectively (Fig. 4h, i), in breast tissue, which is in line with a model of RANKL and RANK being specifically expressed in mature luminal and luminal progenitor cells, respectively. The mean level of RANKL and RANK expression was significantly associated with the *WID-Breast29* index (Fig. 4j).

### Impact of mifepristone on DNA methylation surrogate markers

**Fig. 5:**
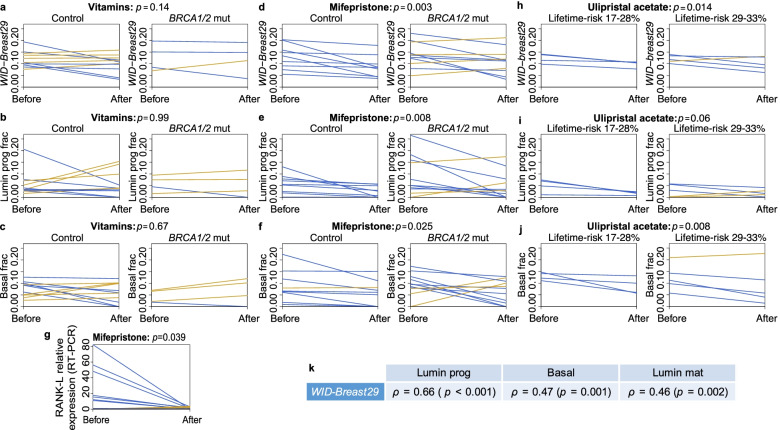
Effects of vitamins (placebo/comparator), mifepristone and ulipristal acetate treatment on intermediate cancer surrogate endpoints in normal breast tissue from healthy women. The impact of Vitamin (**a**–**c**), Mifepristone (**d**–**f**) and Ulipristal acetate (**h**–**j**) on the mitotic age index *WID-Breast29* (**a**, **d**, **h**), luminal progenitor cell fraction (**b**, **e**, **i**) and basal cell fraction (**c**, **f**, **j**) in healthy, unaffected women at lower and higher breast cancer risk. **g** RANKL mRNA expression before and after treatment with mifepristone (as assessed by real-time PCR). **k** Correlation of the change in *WID-Breast29* index with the change in breast epithelial subtype fractions in all samples in **a–f, h–j**. All significances in **a–j** were assessed with the paired-sample *t*-test. Significance in k was assessed with Pearson’s correlation test. Gold lines indicate increasing and blue lines decreasing values from the breast biopsy taken before to biopsy taken after treatment

Next, we assessed whether the progesterone antagonist mifepristone can modulate the epigenetic surrogate markers for replicative age and luminal progenitor cell proportion. We tested the performance of the *WID-Breast29* index in conjunction with the epithelial cell-subtype deconvolution tool in order to evaluate the success of mifepristone therapy in reducing cancer risk. We examined 15 *BRCA* mutation carriers and 20 control women before and after 2-3 month treatment with either vitamins (4 *BRCA* carriers and 11 controls) or mifepristone (11 *BRCA* carriers and 9 controls), respectively (Clinical Trials 1 and 2 *‘Mifepristone treatment prior to insertion of a levonorgestrel releasing intrauterine system for improved bleeding control – a randomized controlled trial’* and *‘The effect of a progesterone receptor modulator on breast tissue in women with BRCA1 and 2 mutations’*; DNAme Set 3; Additional file 1: Fig. S3-4, Table S1b). Each woman provided a breast biopsy before and after taking the study medication. Whereas the *WID-Breast29* index score did not differ amongst women receiving vitamins (Fig. 5a), it was significantly lower after the treatment period amongst women receiving mifepristone (*p*=0.003; Fig. 5d). Whilst all nine control volunteers (100%) showed a reduction in the *WID-Breast29* after mifepristone treatment, this was only seen in 7 of 11 (64%) of the *BRCA* mutant volunteers (Fig. 5d). In two *BRCA1* and two *BRCA2* mutation carriers, mifepristone did not lead to a reduction in the *WID-Breast29* index, possibly indicating adoption of progesterone-independence resulting in lack of response to mifepristone.

Whereas the luminal progenitor cell fraction was not altered by the vitamin intake (Fig. 5b), the vast majority (17 of the 20 (85%) women in the mifepristone group) exhibited a highly significant reduction in the luminal progenitor cell fraction regardless of *BRCA* mutation carrier state (*p*=0.008; Fig. 5e). The decrease in luminal progenitor fraction in the mifepristone group included all nine control volunteers (100%) but only 8 of 11 (73%) of the *BRCA* mutant carriers. Two of the three carriers who showed no decrease in the fraction of luminal progenitors likewise showed no decrease in their *WID-Breast29* score. Finally, whereas vitamins had no impact on the basal cell fraction (Fig. 5c), mifepristone also led to a reduced fraction of basal cells (Fig. 5f) not accompanied by any change in the mature luminal cell fraction (Additional file 1: Fig. S6).

A significant decrease in luminal progenitors was also confirmed from estimates of cell-type proportions based on RNA-seq data (Additional file 1: Fig. S7). The significance of the decrease in luminal progenitor proportion was lower than the decreases estimated based on epigenetic biomarkers illustrating the additional challenges of estimating tissue cell-type composition from data derived from RNA rather than DNA.

Progesterone exerts carcinogenic potential by triggering the expression of RANKL, which leads to the expansion of luminal progenitor cells [23–25, 27]. We used quantitative real-time RT-PCR to show further that expression of RANKL mRNA was reduced to undetectable levels in 15 human volunteers after treatment with the progesterone antagonist mifepristone (Fig. 5g and Additional file 1: Table S5).

### Impact of ulipristal acetate on DNA methylation surrogate markers

In order to assess whether an alternative progesterone antagonist, ulipristal acetate, has a similar impact on the breast we studied breast biopsies from 9 premenopausal volunteers who were judged to have a ≥17% breast cancer lifetime risk and were less than 38 years old at the time of the study, (Clinical Trial 3 ‘*A pilot prevention study of the effects of the anti- progestin Ulipristal Acetate (UA) on surrogate markers of breast cancer risk*’; DNAme Set 4; Additional file 1: Fig. S5), in line with the age-range of the mifepristone trial (Clinical Trial 2). Volunteers responded to ulipristal acetate in a similar way as to mifepristone, showing a significant or borderline-significant reduction in the *WID-Breast29* index (Fig. 5h), the luminal progenitor fraction (Fig. 5i), and the fraction of basal cells (Fig. 5j). Consistent with the mifepristone data, the women whose *WID-Breast29* index increased after ulipristal acetate were in the higher risk group.

The *WID-Breast29* epigenetic index, reflective of the mitotic age, is most significantly correlated with changes in luminal progenitor fraction within the epithelial compartment (Fig. 5k). We hypothesise that antiprogestins preferentially reduce the number of luminal progenitor cells with a high replicative age, which we presume to have the highest cancer risk.

### Impact of antiprogestins on Ki67 DNAme signature

Antiprogestins have been found to reduce the proportion of proliferating cells assessed by Ki67 expression [46, 47]. Using a well-validated method [48], we derived the Ki67 index (*KI-idx*), a DNAme-based signature reflective of accumulated endogenous Ki67 expression (Additional file 1). We confirmed that the Ki67 index is reduced by both mifepristone and ulipristal acetate (Additional file 1: Fig S9). A lower level of cellular proliferation over time within a tissue (inferred from lower overall exposure to endogenous Ki67) would be expected to correspond to lower stem-cell replicative age of that tissue. This is confirmed as change in *WID-Breast29* is strongly reflected by change in *KI-idx* under interventions (Additional file 1: Fig S10). Compared to *WID-Breast29*, the *KI-idx* tends to be underenriched for island and enriched for open sea CpGs following interventions (Additional file 1: Fig S10). Direct comparison of the ulipristal acetate-triggered percentage change of the immunohistochemically-assessed Ki67-positive cells and the *KI-idx* change demonstrates that in 2/3 of the cases the directional changes are the same (Additional file 1: Fig S11).

### Impact of mifepristone on *TP53* mutations

**Fig. 6:**
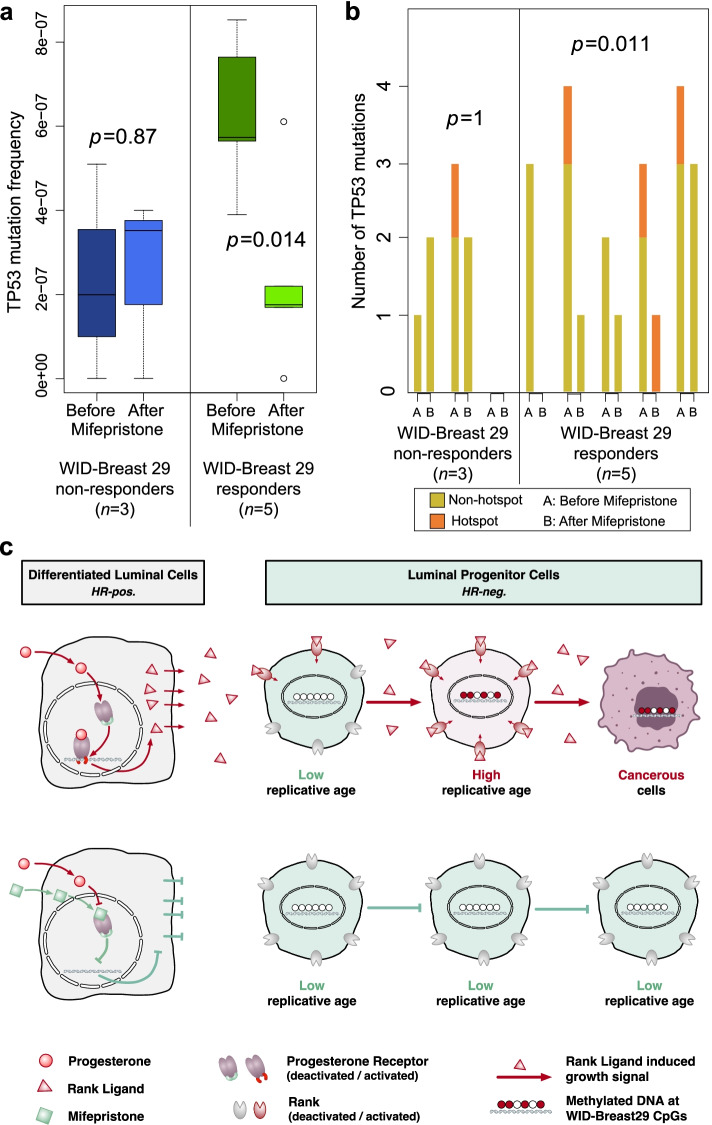
Mifepristone treatment and the dynamics of *TP53* mutations in normal breast tissue and suggested model for the prevention of triple negative breast cancer. **a** *TP53* mutation frequency and **b** *TP53* mutation count in normal breast tissue before and after mifepristone exposure in women who showed a decrease of the *WID-Breast29* index after mifepristone (responders) and who did not show a decrease (non-responders); significance was assessed with the two-sided *t*-test. **c** Progesterone triggers release of RANKL in hormone receptor (HR) positive mature luminal cells leading to increased proliferation and thus accelerated mitotic ageing in HR negative luminal progenitor cells resulting in increased cancer risk; these effects are reduced after treatment with the progesterone antagonist mifepristone. HR, hormone receptor

Somatic *TP53* mutations are highly prevalent in TNBCs [13]. Their presence was also recently documented in a variety of normal tissues [49–53] including the breast [54]. In order to test whether the *TP53* mutation frequency changes as a function of exposure to mifepristone, we applied ultra-accurate duplex sequencing to samples from those volunteers of Clinical Trial 2 (‘*The effect of a progesterone receptor modulator on breast tissue in women with BRCA1 and 2 mutations*’) which we deemed to respond to mifepristone based on the reduction in the *WID-Breast29* index (DNAme Set 3) [55]. This was carried out in eight volunteers treated with mifepristone, where sufficient DNA (≥500 ng) was available before and after treatment. *TP53* mutations were detected in all but one volunteer. The average *TP53* mutation frequency was not significantly different before and after treatment in women who did not respond to mifepristone but was significantly decreased in women who showed a decrease in the mitotic age *WID-Breast29* index in response to mifepristone (Fig. 6a). Although we were not able to detect the same *TP53* clone in any of the pairs, both the number of *TP53* mutations overall, as well as the number of mutations that are known to be hotspot mutations in breast cancer, decreased after mifepristone exposure (Fig. 6b).

## Discussion

Here we demonstrated that women with a *BRCA* mutation have aberrant levels of sex steroid hormones associated with intensified progesterone dominance in the luteal phase of the menstrual cycle (*Hormonal Saliva Data-Set*). Furthermore, we demonstrated that antagonising progesterone with the antiprogestins mifepristone or ulipristal acetate reduces both a surrogate marker for replicative (mitotic) age and the proportion of luminal progenitor cells (both of which are consistently increased in cancer compared to the adjacent normal tissue), as well as the fraction of basal cells (Clinical Trials 1–3 “*Mifepristone treatment prior to insertion of a levonorgestrel releasing intrauterine system for improved bleeding control – a randomized controlled trial’*, *‘The effect of a progesterone receptor modulator on breast tissue in women with BRCA1 and 2 mutations*” and *‘A pilot prevention study of the effects of the anti- progestin Ulipristal Acetate (UA) on surrogate markers of breast cancer risk’*, DNAme Sets 3-4). This and the observations that replicative age is also an independent poor prognostic factor when assessed in cancer tissue and that mifepristone, reduces the number of *TP53* mutant clones, strongly supports the view that the antiprogestins mifepristone and ulipristal acetate are attractive cancer-preventive candidates in young women. Our data implies (subject to confirmation by single-cell sequencing) that antagonising progesterone in young women selectively reduces the pool of those luminal progenitor cells that have reached a high mitotic age and that the efficacy of the preventive measures can be monitored using DNAme markers (Fig. 6c/d), which can alternatively be assessed by real-time PCR (Additional file 1: Fig S8).

Our data align with previous findings that indicate that an intrinsic defect exists in one or more signalling pathways in *BRCA1*-deficient luminal progenitor cells, allowing them to bypass the requirement for exogenous factors like progesterone [56]; we postulate that this “intrinsic defect” might have been triggered by aberrant hormonal exposure over several decades prior to obtaining the luminal progenitor cells for experimental analyses.

Although our study is limited by the available number of healthy volunteers treated with antiprogestins (Clinical Trials 1-3, ‘*Mifepristone treatment prior to insertion of a levonorgestrel releasing intrauterine system for improved bleeding control – a randomized controlled trial*’, ‘*The effect of a progesterone receptor modulator on breast tissue in women with BRCA1 and 2 mutations*’ and ‘*A pilot prevention study of the effects of the anti- progestin Ulipristal Acetate (UA) on surrogate markers of breast cancer risk*’; DNAme Sets 3-4), our data lend support for prospective non-surgical cancer risk reduction trials in *BRCA1/2* mutation carriers which can integrate the *WID-Breast29* test in conjunction with assessment of cellular composition of mammary epithelium as a means of monitoring patient responsiveness. We note that changes in mammographic breast density, an established good predictor of response to tamoxifen in cancer prevention studies [57], may also be useful as a surrogate biomarker of responsiveness in cancer risk reduction trials. However, we favour the epigenetic biomarkers described here, at least in young premenopausal high-risk women, because (i) they are an excellent specific surrogate of epithelial cells at risk, (ii) they can be serially measured at frequent intervals, (iii) the dynamic of the *WID-Breast29* in individual volunteers reflects cancer risk in real time allowing for immediate adjustments, and (iv) these measurements can be obtained using minimally invasive procedures not dependent on repeated exposure to X-rays.

Our results also suggest that progesterone signalling could be a suitable target to achieve non-surgical cancer risk reduction in high-risk populations. This could potentially be achieved by antagonising RANKL, a downstream mediator of progesterone signalling in the breast that can prevent cancer in rodents [25]. Denosumab, a fully humanised monoclonal antibody that binds RANKL and reduced breast epithelial proliferation in three premenopausal volunteers, is already available and used in the treatment of osteoporosis [58]. However, this agent has no demonstrable impact on the incidence of contralateral breast cancer in postmenopausal women [59]. Progesterone has been shown to trigger early-onset cancer dissemination [2] and hence antagonising progesterone signalling may be more effective than blocking RANKL, also given that progesterone not only increases mammary RANKL levels but also reduces circulating levels of the systemic RANKL-antagonist OPG [22]. Mifepristone is a progesterone receptor antagonist [60] currently used for pregnancy termination [61, 62] and as emergency contraceptive [63]. It has been extensively studied for the treatment of uterine fibroids [64], endometriosis [65] and as a long term contraceptive [66]. It exerts an antiproliferative effect in breast tissues of healthy women [46] and reduces mammary cancer development in mice [29], further attesting to its potential merit as a cancer risk-reducing agent in germline *BRCA1/2* mutation carriers.

Although the efficacy of mifepristone and ulipristal acetate cannot be directly compared in our setting as the two trials recruited different groups of volunteers, the observation that both progesterone antagonists but not vitamins reduced the luminal progenitor and basal cell fractions (i.e. breast epithelial cell-types which also depend on progesterone [11]), indicates that both drugs exert a progesterone-antagonistic effect in the breast. In our studies, antiprogestins were only given to premenopausal women less than 38 years old; whether antiprogestins would be similarly effective in premenopausal women over 38 years, or postmenopausal women, needs to be determined.

Currently, the most effective means of cancer prevention in *BRCA1/2* mutation carriers entails surgical removal of the organs at risk. A significant number of individuals chose to postpone these potentially life-saving surgeries due to the adverse effects on fertility and the social/emotional side effects. It is therefore vitally important that effective non-surgical means of cancer risk reduction be developed. This is especially relevant to young and otherwise healthy individuals who unexpectedly learn that a close relative carries a *BRCA1* or *BRCA2* mutation subsequently documented in their own germline. This underscores the potential impact of a non-surgical risk reduction trial such as proposed here. Unlike tamoxifen, which has been assessed in millions of breast cancer patients (in the adjuvant setting) before it has been studied in healthy women for primary breast cancer prevention, no sufficient experience exists yet for long-term treatment with antiprogestins. Aspects of liver toxicity (described for ulipristal acetate [67] but not for mifepristone) as well as the impact of long term treatment of antiprogestins on the endometrium and immune function will need to be assessed prior to considering selective progesterone receptor modulators in a breast and ovarian cancer-preventive setting. Certainly, entirely novel strategies (i.e. drug holidays and monitoring molecular markers like the ones we have described here using tissue or cells from a breast biopsy of fine-needle aspirates) will have to be explored.

## Conclusions

Progesterone drives expansion of luminal progenitor cells, thought to be the cell-of-origin of breast tumours with the worst prognosis. Hence, we studied whether antagonising progesterone can prevent breast cancers. We found daily levels of progesterone to be higher throughout the menstrual cycle of *BRCA1/2* mutation carriers, implying progesterone signalling may be targeted for cancer risk reduction in this population. Breast field cancerization DNA methylation signatures quantifying replicative age and luminal progenitor cell numbers were increased in breast cancers (which are highly prevalent in *BRCA* carriers), which strongly suggests that luminal progenitors with elevated replicative age are more prone to malignant transformation. The progesterone receptor antagonist mifepristone reduced both the mitotic age and the proportion of luminal progenitor cells in breast tissue, and these findings were validated by an alternate progesterone receptor antagonist, ulipristal acetate. Intriguingly, mifepristone reduced both the *TP53* mutation frequency as well as the number of *TP53* mutations in mitotic-age-responders, suggesting a possible mechanism behind the observed cancer risk reduction. Therefore, these data provide the best evidence to date for the potential usage of antiprogestins for primary prevention of poor-prognostic breast cancers.

## Supplementary Information

**Additional file 1: Supplementary Methods.** **Fig. S1.** Elevated levels of estradiol and progesterone in *BRCA1* and *BRCA2* mutation carriers compared to controls. **Fig. S2.** Epithelial cell composition in normal tissues adjacent to TNBC compared to cancer-free breasts. **Fig. S3.** Enrolment and randomisation in Clinical Trial 1. **Fig. S4.** Enrolment and randomisation in Clinical Trial 2. **Fig. S5.** Enrolment in Clinical Trial 3. **Fig. S6.** Effect of mifepristone treatment on breast epithelium cell subtype composition. **Fig. S7.** Effect of mifepristone treatment on breast epithelium cell subtype composition – RNA-seq data. **Fig. S8.** The *WID-Breast5* index. **Fig. S9.** The Ki67 index. **Fig. S10.** Comparing the Ki67 index with the *WID-Breast29* index. **Fig. S11.** Comparing the change (before and after ulipristal acetate) in percentage of immunohistochemistry Ki67-positive cell with change in Ki67-index in DNAme Dataset 4 (Clinical trial 3, ‘*A pilot prevention study of the effects of the anti- progestin Ulipristal Acetate (UA) on surrogate markers of breast cancer risk’*). **Table S1a.** Summary statistics about the 20 healthy women (12 *BRCA1* mutation carriers and 8 *BRCA1* wild type) enrolled in the trial to measure estradiol and progesterone levels throughout the menstrual cycle (see also Figs. 1 and 2). **Table S1b.** Summary statistics about the 21 healthy *BRCA* mutation carriers enrolled in Clincal Trial 2 who provided samples for DNAme analysis as part of DNAme Set 3. **Table S2.** The 37 *WID-Breast29* CpGs. **Table S3.** Summary statistics about the donors of the breast cancer biopsies and matched surrounding normal tissue samples. **Table S4.** The reference CpGs identified for luminal progenitor, mature luminal, and basal breast epithelial cells. **Table S5.** Summary statistics about the 15 healthy women who provided samples for RT-PCR and RNA-seq before and after mifepristone treatment.

**Additional file 2.** Is the protocol for Clinical Trial 1, *‘Mifepristone treatment prior to insertion of a levonorgestrel releasing intrauterine system for improved bleeding control – a randomized controlled trial’* (EudraCT registration number 2009-009014-40).

**Additional file 3.** Is the protocol for Clinical Trial 2, *‘The effect of a progesterone receptor modulator on breast tissue in women with BRCA1 and 2 mutations’* (EudraCT registration number: 2012-003703-35).

**Additional file 4.** Is the protocol for Clinical Trial 3, *‘A pilot prevention study of the effects of the anti- progestin Ulipristal Acetate (UA) on surrogate markers of breast cancer risk’* (EudraCT registration number: 2015-001587-19).

## Data Availability

The DNA methylation data, demographics, and cancer treatment information generated and analysed in this study as DNAme Sets 1 and 3 are accessible at the EGA website under the accession number EGAS00001005070 (https://ega-archive.org/studies/EGAS00001005070) [68]. The DNA methylation data, demographics, and cancer treatment information generated and analysed in this study as DNAme Set 2 are accessible at the NCBI GEO website under the accession number GSE69914 (https://www.ncbi.nlm.nih.gov/geo/query/acc.cgi?acc=GSE69914) [69]. The DNA methylation data, demographics, and cancer treatment information generated and analysed in this study as DNAme Set 4 are accessible at the NCBI GEO website under the accession number GSE201724 (https://www.ncbi.nlm.nih.gov/geo/query/acc.cgi?acc=GSE201724) [70]. The RNA sequencing data, demographics, and cancer treatment information generated and analysed in this study are accessible at the NCBI GEO website under the accession number GSE157126 (https://www.ncbi.nlm.nih.gov/geo/query/acc.cgi?acc=GSE157126) [71].
