# Correction: Antiprogestins reduce epigenetic field cancerization in breast tissue of young healthy women

**DOI:** 10.1186/s13073-022-01086-y

**Published:** 2022-07-19

**Authors:** Thomas E. Bartlett, Iona Evans, Allison Jones, James E. Barrett, Shaun Haran, Daniel Reisel, Kiriaki Papaikonomou, Louise Jones, Chiara Herzog, Nora Pashayan, Bruno M. Simões, Robert B. Clarke, D. Gareth Evans, Talayeh S. Ghezelayagh, Sakthivignesh Ponandai-Srinivasan, Nageswara R. Boggavarapu, Parameswaran G. Lalitkumar, Sacha J. Howell, Rosa Ana Risques, Angelique Flöter Rådestad, Louis Dubeau, Kristina Gemzell-Danielsson, Martin Widschwendter

**Affiliations:** 1 Department of Statistical Science, University College London, London, WC1E 7HB UK; 2 Department of Women’s Cancer, UCL EGA Institute for Women’s Health, University College London, 74 Huntley Street, London, WC1E 6AU UK; 3 European Translational Oncology Prevention and Screening (EUTOPS) Institute, Universität Innsbruck, 6060 Hall in Tirol, Austria; 4 Research Institute for Biomedical Aging Research, Universität Innsbruck, 6020 Innsbruck, Austria; 5 Department of Women’s and Children’s Health, Division of Obstet- rics and Gynecology, Karolinska Institutet and Karolinska University Hospital, Stockholm, Sweden; 6 Centre for Tumour Biology Department, Barts Cancer Institute, Queen Mary University of London, London, UK; 7 Department of Applied Health Research, University College London, 1-19 Torrington Place, London, WC1E 7HB UK; 8 Breast Biology Group, Manchester Breast Centre, Divi- sion of Cancer Sciences, Faculty of Biology, Medicine and Health, University of Manchester, Manchester, UK England; 9 University of Manchester, St. Mary’s Hospital, and University Hospital of South Manchester, Manchester, UK; 10 Department of Laboratory Medicine and Pathology, University of Washing- ton, Seattle, WA 98195 USA; 11 Department of Obstetrics and Gynecology, University of Washington, Seattle, WA 98195 USA; 12 Department of Medical Oncology, The Christie NHS Foundation Trust, Manchester, UK; 13 Department of Pathology, Keck School of Medicine, USC/Norris Comprehensive Cancer Centre, University of Southern California, Los Angeles, USA

**Correction: Genome Med 14, 64 (2022)**

**https://doi.org/10.1186/s13073-022-01063-5**

Following publication of the original article [1], an error was identified in the Funding section.

The section currently reads:

This study was funded by the European Union’s Horizon 2020 European Research Council Programme, H2020 BRCA-ERC under Grant Agreement No. 742432 as well as the charity The Eve Appeal (https://eveappeal.org.uk). Additional aspects of the study were supported by the Swedish Research Council Grant # 2017-00932, 2012-02961, Region Stockholm and Karolinska Institutet (ALF), Cancerfonden (Grant no. CAN 2016/696), and Radiumhem- mets Forskningsfonder (# 154143, 184033) as well as by the NIHR Manchester Biomedical Research Centre (IS-BRC-1215-20007). The work carried out by TEB was supported by MRC grant MR/P014070/1.

It should read:

This study was funded by the European Union’s Horizon 2020 European Research Council Programme, H2020 BRCA-ERC under Grant Agreement No. 742432 as well as the charity The Eve Appeal (https://eveappeal.org.uk). Additional aspects of the study were supported by the Swedish Research Council Grant # 2017-00932, 2012-02961, Region Stockholm and Karolinska Institutet (ALF), Cancerfonden (Grant no. CAN 2016/696), and Radiumhem- mets Forskningsfonder (# 154143, 184033) as well as by the NIHR Manchester Biomedical Research Centre (IS-BRC-1215-20007). The work carried out by TEB was supported by MRC grant MR/P014070/1. Clinical trial 3 was supported by Breast Cancer Now [Grant Ref no: 2014May-CR003]”

The original article [1] has been corrected.
