# Supplementary material for: Antiprogestins reduce epigenetic field cancerization in breast tissue of young healthy women
Source: Genome Med. 2022 Jun 15;14:64. doi: 10.1186/s13073-022-01063-5 (PMC9199133; doi:10.1186/s13073-022-01063-5)
Supplement: Supplementary file 1 — Additional file 1: Supplementary Methods. Fig. S1. Elevated levels of estradiol and progesterone in BRCA1 and BRCA2 mutation carriers compared to controls. Fig. S2. Epithelial cell composition in normal tissues adjacent to TNBC compared to cancer-free breasts. Fig. S3. Enrolment and randomisation in Clinical Trial 1. Fig. S4. Enrolment and randomisation in Clinical Trial 2. Fig. S5. Enrolment in Clinical Trial 3. Fig. S6. Effect of mifepristone treatment on breast epithelium cell subtype composition. Fig. S7. Effect of mifepristone treatment on breast epithelium cell subtype composition – RNA-seq data. Fig. S8. The WID-Breast5 index. Fig. S9. The Ki67 index. Fig. S10. Comparing the Ki67 index with the WID-Breast29 index. Fig. S11. Comparing the change (before and after ulipristal acetate) in percentage of immunohistochemistry Ki67-positive cell with change in Ki67-index in DNAme Dataset 4 (Clinical trial 3, ‘A pilot prevention study of the effects of the anti- progestin Ulipristal Acetate (UA) on surrogate markers of breast cancer risk’). Table S1a. Summary statistics about the 20 healthy women (12 BRCA1 mutation carriers and 8 BRCA1 wild type) enrolled in the trial to measure estradiol and progesterone levels throughout the menstrual cycle (see also Figs. 1 and 2). Table S1b. Summary statistics about the 21 healthy BRCA mutation carriers enrolled in Clincal Trial 2 who provided samples for DNAme analysis as part of DNAme Set 3. Table S2. The 37 WID-Breast29 CpGs. Table S3. Summary statistics about the donors of the breast cancer biopsies and matched surrounding normal tissue samples. Table S4. The reference CpGs identified for luminal progenitor, mature luminal, and basal breast epithelial cells. Table S5. Summary statistics about the 15 healthy women who provided samples for RT-PCR and RNA-seq before and after mifepristone treatment. [file 13073_2022_1063_MOESM1_ESM.pdf]

SUPPLEMENTARY INFORMATION

Antiprogestins reduce epigenetic field cancerization in breasts of young healthy women

Thomas E. Bartlett, Ph.D., Iona Evans, Ph.D., Allison Jones, B.Sc., James E. Barrett, Ph.D., Shaun Haran, M.D., Daniel Reisel, Ph.D., Kiriaki Papaikonomou, M.D., Louise Jones, Ph.D., Chiara Herzog, Ph.D., Nora Pashayan, PhD, M.D., Bruno M Simões, Ph.D., Robert B Clarke, Ph.D., D. Gareth Evans, M.D., Talayeh S Ghezelayagh, Ph.D., Sakthivignesh Ponandai-Srinivasan, Ph.D., Nageswara R Boggavarapu, Ph.D., Parameswaran G Lalitkumar, Ph.D., Sacha J Howell, M.D., Ph.D., Rosa Ana Risques, M.D., Ph.D., Angelique Flöter Rådestad, M.D., Louis Dubeau, M.D., PhD, Prof Kristina Gemzell-Danielsson, M.D. and Prof Martin Widschwendter M.D.*.

SUPPLEMENTARY METHODS:

Hormone Analyses using Enzyme Immunoassay

Salivary hormone levels were measured using enzyme immunoassay kits from Salimetrics; for Progesterone (#1-1502-5) and Estradiol (#1-4702-5), following the manufacturer's instructions. Briefly, saliva samples were thawed overnight at 4ºC, mixed by inverting, and centrifuged for 15 minutes at 1500rpm. 50µl (for progesterone (P)) or 100µl (for Estradiol (E)) of sample was placed onto a microtitre assay plate and HRP-enzyme conjugate added. Plates were shaken (500rpm) and incubated at room temperature for 1 (P) or 2 (E) hours, then washed 4 times with 1x Wash buffer. Tetramethylbenzidine (TMB) substrate was added, the plate shaken and then incubated for 30 minutes in the dark. Stop solution was added before plates were briefly shaken and read at 450nm on a Varioskan-LUX plate reader. A standard curve

was run on each plate. Data was analysed and hormone levels calculated by interpolation using a 4-paramater non-linear regression curve fit in Graphpad Prism.

DNA methylation data processing

All DNAme data were background-corrected and normalised with BMIQ [2]. Probes were removed if they had < 95% coverage across samples and any remaining probes with detection *p*-value > 0.05 were replaced by *k*-NN imputation, with *k*=5. The same quality control and normalisation procedure was followed for 257 breast cancer invasive carcinoma samples with associated clinical data, and 216 breast cancer invasive carcinoma samples and 38 healthy control breast tissue samples with matched gene-expression data, that are publicly available from the TCGA (The Cancer Genome Atlas) repository (https://www.cancer.gov/about-nci/organization/ccg/research/structural-genomics/tcga).

Bisulphite-sequenced DNAme data for purified breast epithelial cell subtypes were downloaded from the European Genome-Phenome Archive (EGA) under accession number EGAS00001000552 [S1] and reads were aligned and counted using Bismark [3] with default settings. We subsequently retained only reads mapping to CpGs represented on the Illumina EPIC array and which had a total number of mapped reads (methylated+unmethylated) of at least 20.

Gene expression analysis

Gene-expression data from 216 breast tumour and 38 healthy control breast tissue samples with matched DNAme data available were downloaded from the TCGA repository (The Cancer Genome Atlas,
https://www.cancer.gov/about-nci/organization/ccg/research/structural-genomics/tcga,
(*TCGA Matched Gene Expression and DNAme Set*) and were then quantile-normalised using the preprocessCore package in R. The mean normalised expression level of RANK and

RANKL was calculated as $x_m=(x_1+x_2)/2$, where x_1 and x_2 are the normalised expression levels of RANK and RANKL respectively, and x_m is the mean normalised expression level.

RNA-seq data – additional information

Transcriptome data for the same purified breast epithelial cell subtypes as for the DNAme data were also downloaded from the European Genome-Phenome Archive (EGA) under accession number EGAS00001000552. Transcriptome data for purified fat cell samples ENCFF072HRK, ENCFF654JLY, ENCFF732LRY and ENCFF924FNY were downloaded from the ENCODE (Encyclopedia of DNA Elements) repository (https://www.encodeproject.org). Read-counts were obtained from the archived bam files for the RNA-seq libraries (as downloaded from the online repositories) using Samtools featureCounts. TPM-normalised counts were then used for all downstream analyses.

Statistical analysis

The *WID-Breast29* index was defined by selecting a subset of the 385 CpG loci contributing to the *pcgtAge* index[4] (a measure of 'biological mitotic age'). Specifically, we selected those CpG loci from the *pcgtAge* index, which showed increasing methylation levels in normal breast tissue of *BRCA1/2* mutation carriers (n=14) compared to normal breast tissue from controls (n=14). We assessed this increase by the difference in median methylation levels of the samples in these groups for each CpG locus. Reasoning that CpG loci with only very small increases in median DNAme level are probably less informative, we selected only CpGs with median delta beta >0.01, comparing *BRCA1/2* mutation carriers with controls. The result was 37 CpG loci that passed this threshold (Supplementary Table 2). The *WID-Breast29* index is defined as the mean methylation level of these 37 CpGs in a sample.

We also developed a Ki67 index, *KI-idx,* as a surrogate measure of tissue samples' exposure to endogenous Ki67 (Fig. S9). Reasoning that endogenous Ki67 activity leads to, or is at least associated with, changes in DNA methylation levels at certain CpG loci, we sought to detect

these changes and summarise them in a one-number score or index, using a well-validated method [5] as follows. Spearman's correlation test p-values were calculated for each CpG, by correlating its methylation level across 216 breast tumour samples downloaded from TCGA with MKI67 expression level in the same samples. The *KI-idx* is based on the top 4528 CpGs (FDR-adjusted p<0.001, roughly the top 1% of CpGs on the microarray), and is calculated for tissue sample j as:

$$KI\text{-}idx_j = \Sigma_{\, i:q_i<0.001}\, w_i \,(\, \beta_{ij} - \mu_i \,) \,/\, \sigma_i \,,$$

where $w_i = w^*_i / \Sigma w^*_i$, with $w^*_i = -s_i \log10 p_i$, where p_i is the correlation test p-value for CpG i, $s_i = \pm 1$ according to positive or negative correlation, and μ_i and σ_i are the mean and standard-deviations of the methylation levels of CpG i across the 216 tumour samples.

We estimated the composition of each tissue sample in terms of proportions of epithelial, fibroblast, immune and fat cells, based on the DNAme profile of each sample, using a well-validated algorithm [6]. We then developed a new algorithm to further decompose the breast epithelial compartment in terms of basal, luminal progenitor, and mature luminal cells [7]. To do this, we defined a custom DNAme reference profile for each of these epithelial cell subtypes (Supplementary Table 4) based on bisulphite-sequenced DNAme data that have been previously published and are available from the European Genome-Phenome Archive (EGA) under accession number EGAS00001000552. To define these custom DNAme reference profiles, we followed the same procedure that was used to define the original DNAme reference profiles used in the general tissue-decomposition algorithm [6]. Specifically, we required that in the original reference DNAme data-sets for epithelial, fibroblast, immune and fat cells, the beta-values for our selected reference CpGs have variance <0.001, and for fibroblast, immune and fat cells, they have mean beta >0.7 or <0.3. We then required that our selected reference CpGs have a difference in mean beta >0.5 between each of these original reference profiles and those for each of the epithelial subtypes. Finally, we required that our selected reference

CpGs for basal cells have mean beta >0.2 different from each of the other subtypes, for mature luminal cells >0.7 different, and for luminal progenitors >0.45 different from each of the other subtypes. These thresholds vary for each epithelial subtype to ensure that similar numbers of CpGs for each subtype are included in the reference profile. We note that this identified set of epithelial subtype reference CpGs does not overlap at all with the original reference CpGs for epithelial, fibroblast, immune and fat cells that were defined for the general tissue-decomposition algorithm [6]. This breast epithelial cell subtype inference method for DNAme data is available as an R package from: https://github.com/tombartlett/BreastEpithelialSubtypes

We also used transcriptome data to confirm changes we found in the composition of each tissue sample. We did this by estimating tissue samples in terms of the proportions of the epithelial subtypes (luminal progenitor, mature luminal and basal cells), as well as stromal and fat cells (the proportion of immune cells was found to be negligible). We used a well tested and reliable statistical method and implementation for this purpose [8], together with reference RNA-seq profiles from the same purified breast cell subtype data-set as we used for the reference DNAme profiles, with a reference RNA-seq profile for fat estimated from data downloaded from the ENCODE repository.

SUPPLEMENTARY FIGURES

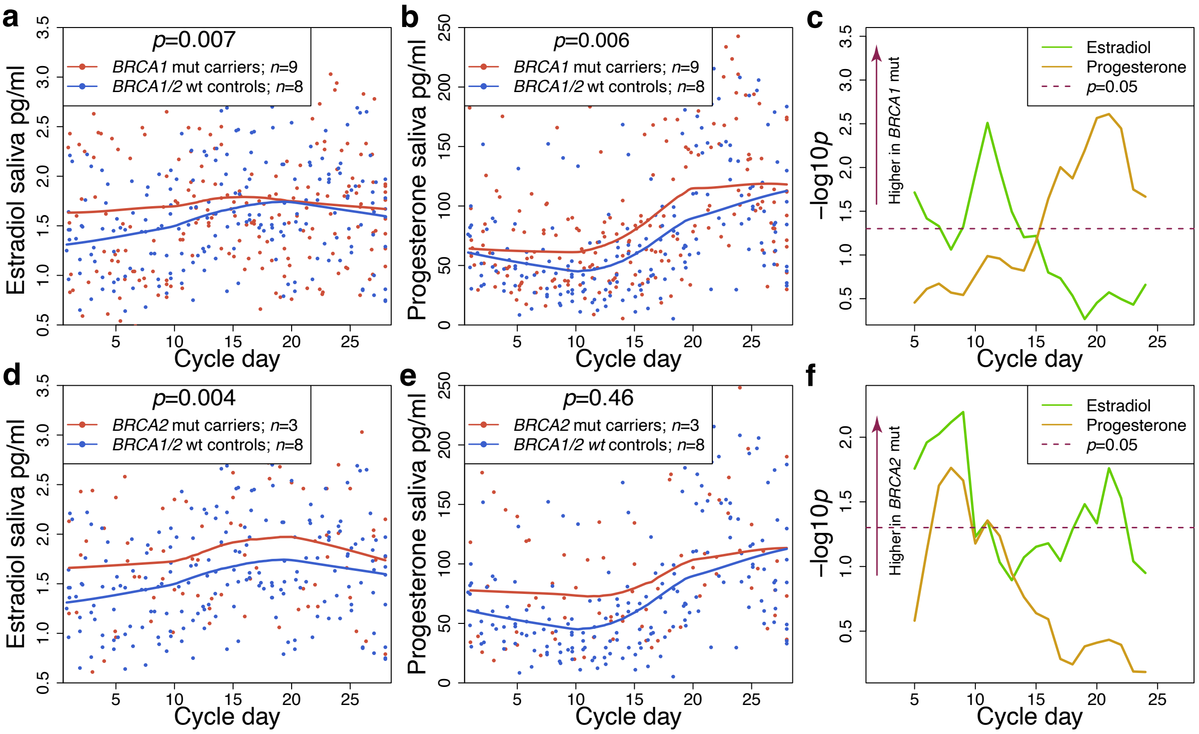

Fig. S1. Elevated levels of estradiol and progesterone in *BRCA1* and *BRCA2* mutation carriers compared to controls.

Estradiol (**a** and **d**) and progesterone (**b** and **e**) hormone levels are compared over one cycle in *BRCA1* (*n*=9, **a** and **b**) and *BRCA2* (*n*=3), **d** and **e** with controls (*n*=8), with best-fit lines. One-week moving windows were used to assess how the significance in increase in hormone levels in *BRCA1* or *BRCA2* mutation carriers (compared to controls) varies during the cycle (**c** and **f** respectively). Significances were calculated with the *t*-test.

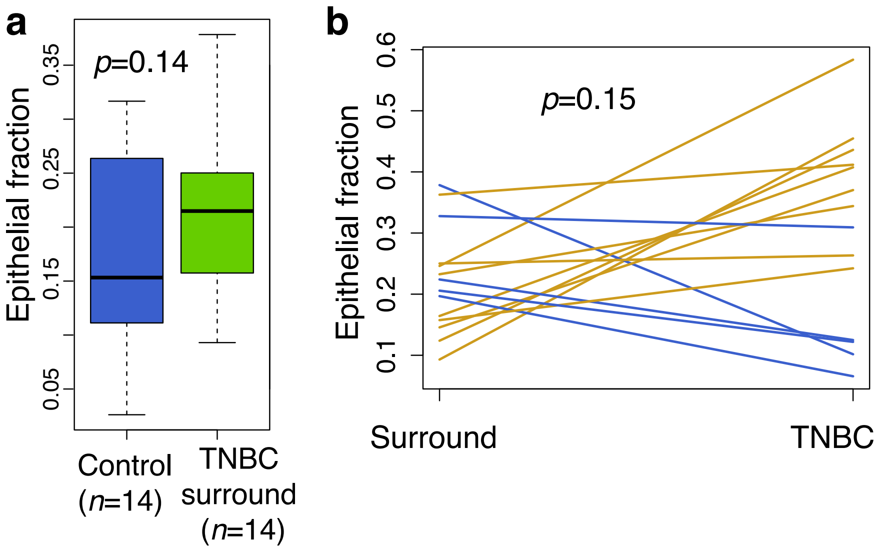

Fig. S2. Epithelial cell composition in normal tissues adjacent to TNBC compared to cancer-free breasts.

a The epithelial cell fraction did not significantly change between normal breast tissue surrounding TNBC (n=14) compared to normal tissue from cancer-free women (n=14), significance was assessed with the t-test. **b** The epithelial cell fraction did not significantly change between normal tissue surrounding TNBC compared to TNBC in the same volunteers (n=14); significance was assessed with the paired-sample t-test.

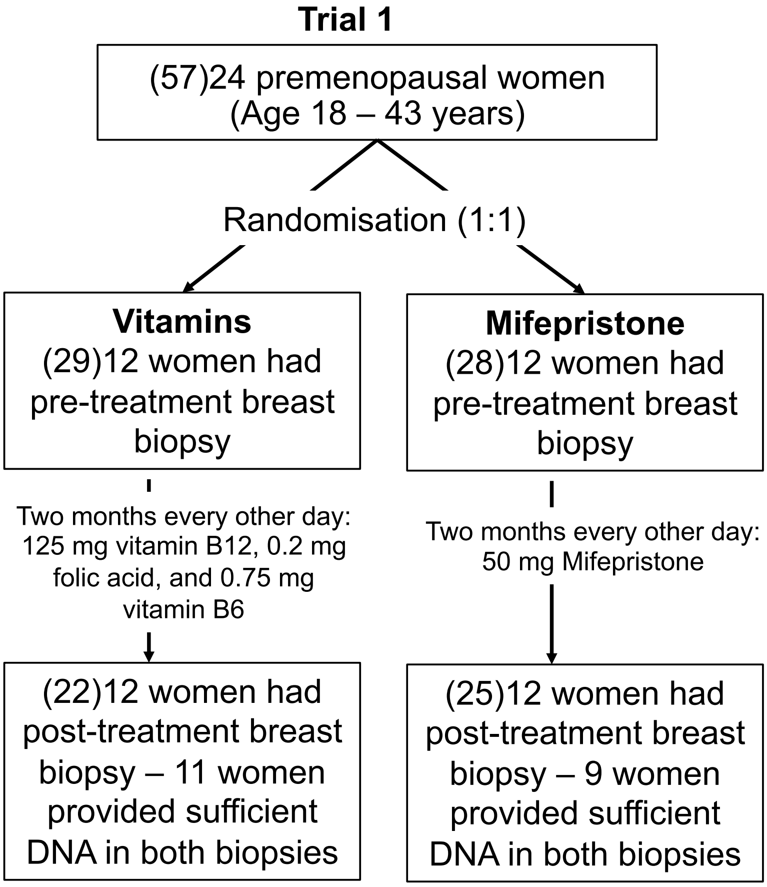

Fig. S3. Enrolment and randomisation in Clinical Trial 1.

CONSORT-style flow-chart summarising Clinical Trial 1, '*Mifepristone treatment prior to insertion of a levonorgestrel releasing intrauterine system for improved bleeding control – a randomized controlled trial'*). Numbers in brackets represent the total number of women recruited. In total, 57 women were recruited, and were randomised 1:1 in mifepristone and vitamins (control) arms. Of these, 12 women in each trial arm had pre- and post- treatment breast biopsies. Those in the mifepristone arm received 50mg mifepristone every other day for two months, whereas those in the vitamins arm received 125mg vitamin B12, 0.2mg folic acid, and 0.75mg vitamin B6 every other day for 2 months. In the mifepristone and vitamin arms, 9 and 11 women (respectively) provided sufficient DNA in both biopsies to carry out subsequent DNAme analysis.

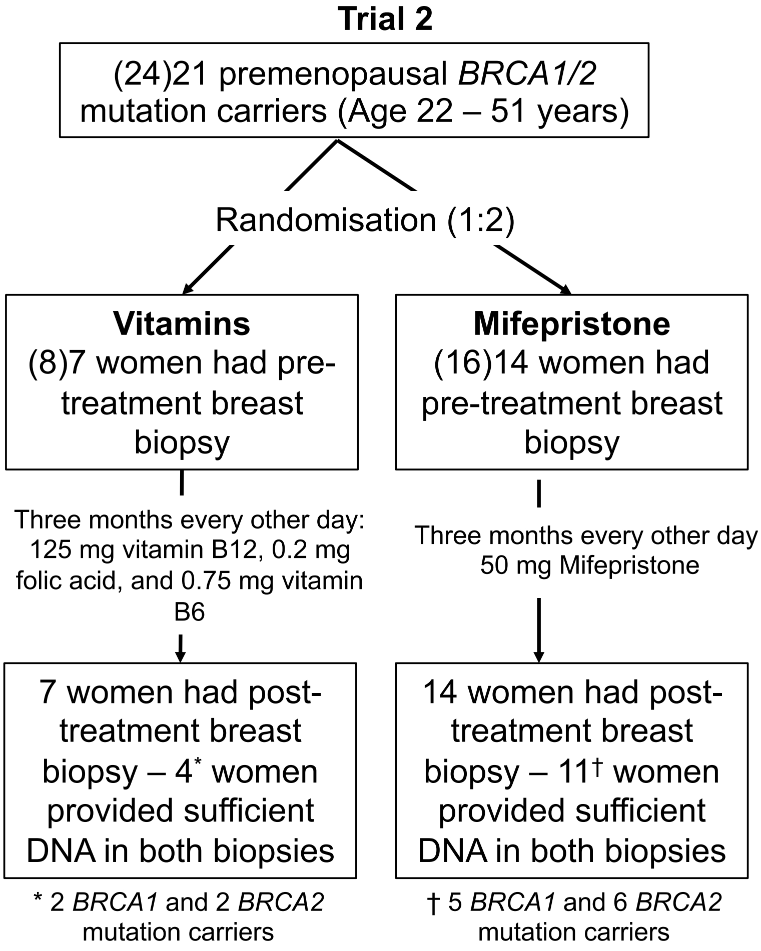

Fig. S4. Enrolment and randomisation in Clinical Trial 2.

CONSORT-style flow-chart summarising Clinical Trial 2, '*The effect of a progesterone receptor modulator on breast tissue in women with BRCA-1 and -2 mutations'*. Numbers in brackets represent the total number of women recruited. In total, 24 women were recruited, and were randomised 2:1 in mifepristone and vitamins (control) arms. Of these, 14 women in the mifepristone arm and 7 women in the vitamins arm had pre- and post- treatment breast biopsies. Those in the mifepristone arm received 50mg mifepristone every other day for three months, whereas those in the vitamins arm received 125mg vitamin B12, 0.2mg folic acid, and 0.75mg vitamin B6 every other day for three months. In the mifepristone and vitamin arms, 11 and 4 women (respectively) provided sufficient DNA in both biopsies to carry out subsequent DNAme analysis.

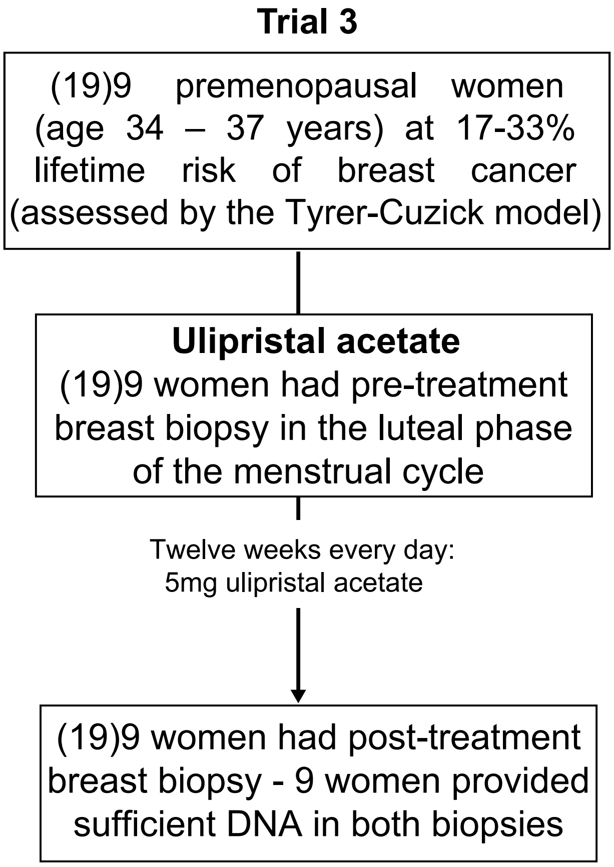

Fig. S5. Enrolment in Clinical Trial 3.

CONSORT-style flow-chart summarising Clinical Trial 3, '*A pilot prevention study of the effects of the anti- progestin ulipristal acetate (UA) on surrogate markers of breast cancer risk*'. Numbers in brackets represent the total number of women recruited. In total, 9 women were recruited, and single ulipristal acetate study arm. Of these, 9 women had pre- and post-treatment breast biopsies, between which they received 5mg ulipristal acetate every day for twelve weeks. All 9 women provided sufficient DNA in both biopsies to carry out subsequent DNAme analysis.

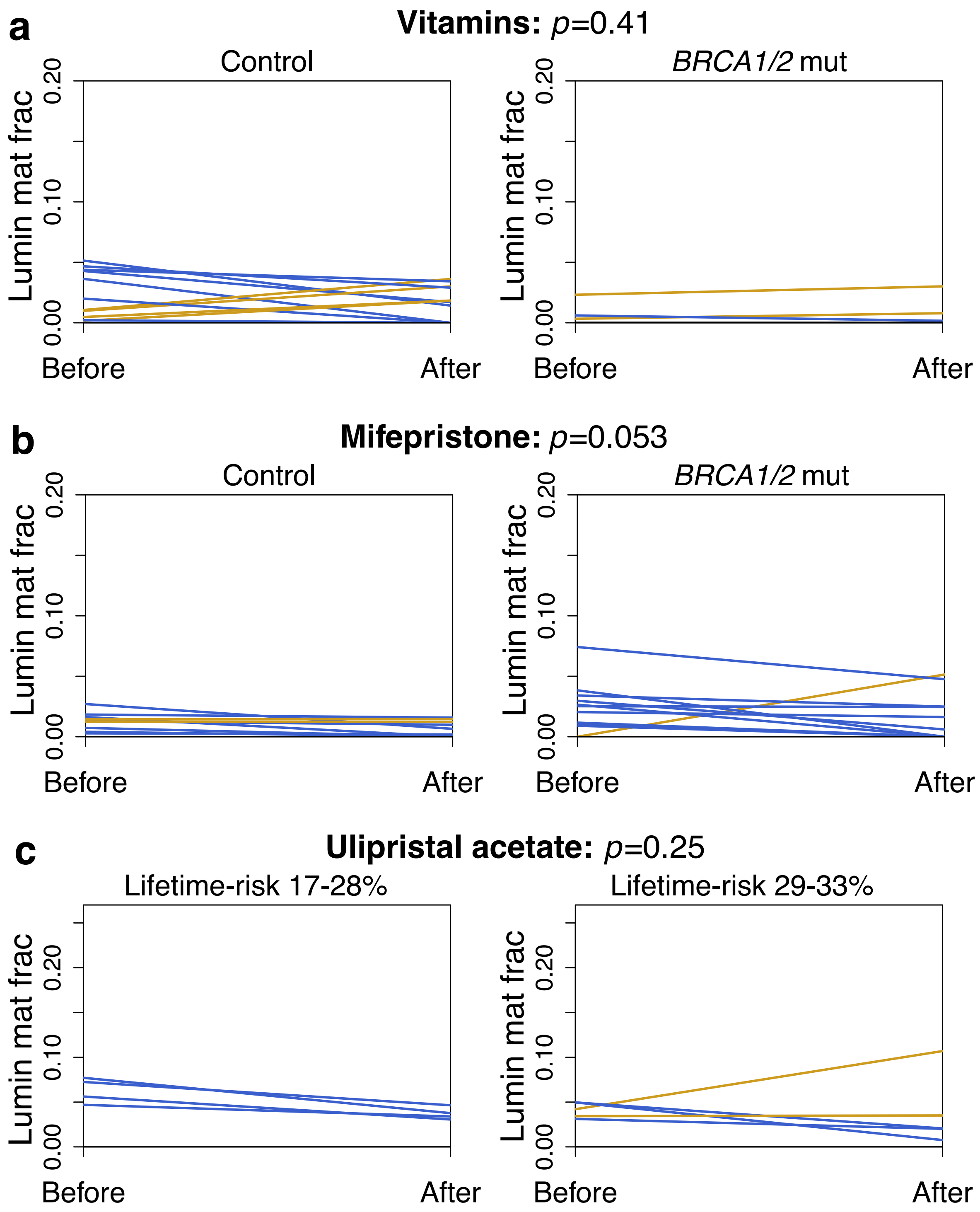

Fig. S6. Effect of mifepristone treatment on breast epithelium cell subtype composition.

a Treatment with vitamins did not significantly reduce the concentration of mature luminal cells, whereas **b** it did decrease with mifepristone (borderline significance), and **c** it did not decrease with ulipristal acetate. Significances were assessed with the paired-sample *t*-test.

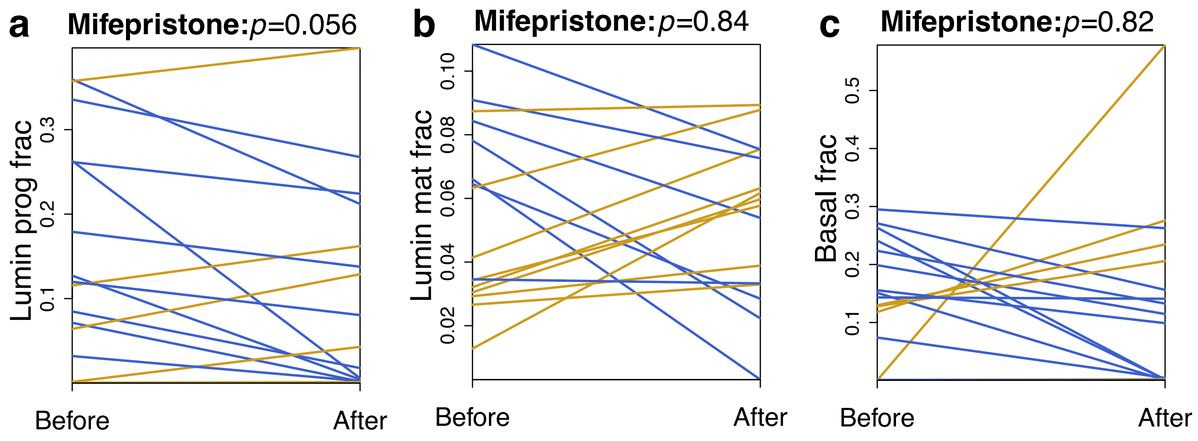

Fig. S7. Effect of mifepristone treatment on breast epithelium cell subtype composition – RNA-seq data.

a Treatment with mifepristone of 15 volunteers (Supplementary Table 5) lead to a borderline-significant decrease in luminal progenitor cell fraction, but did not lead to a significant change in fraction of **b** mature luminal or **c** basal cells. Significances were assessed with the paired-sample *t*-test.

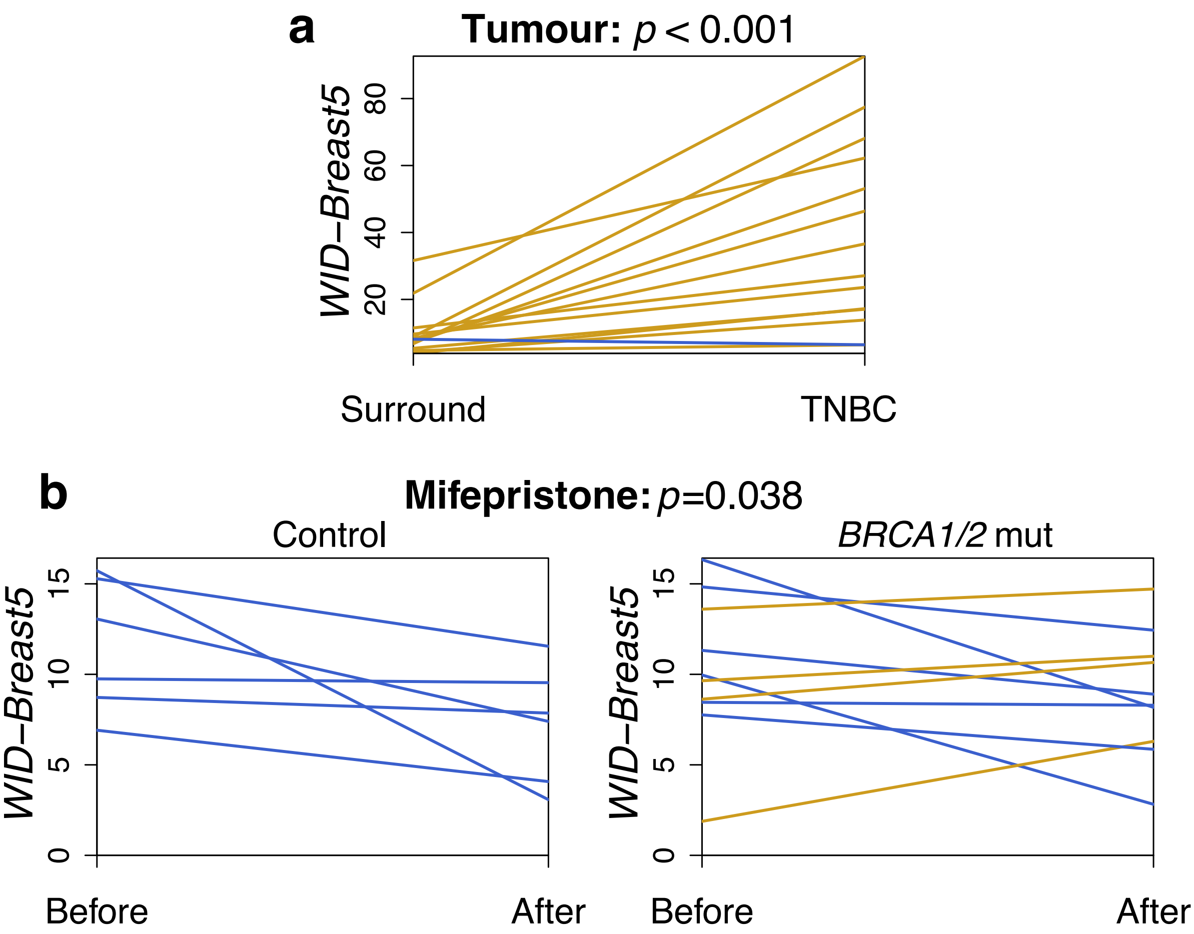

Fig. S8. The *WID-Breast5* index.

The *WID-Breast5* signature (i.e., the summary of percentage of fully methylated reference (PMR) based on MethyLight of the promoter regions for the following 5 genes: MT1A, HOXD12, LHX8, NEUROD1, CBLN4) in **a** normal tissue surrounding triple-negative breast cancer (TNBC) and TNBC itself and **b** before and after mifepristone in control and *BRCA1/2* mutant women. Significances were assessed with the paired-sample *t*-test.

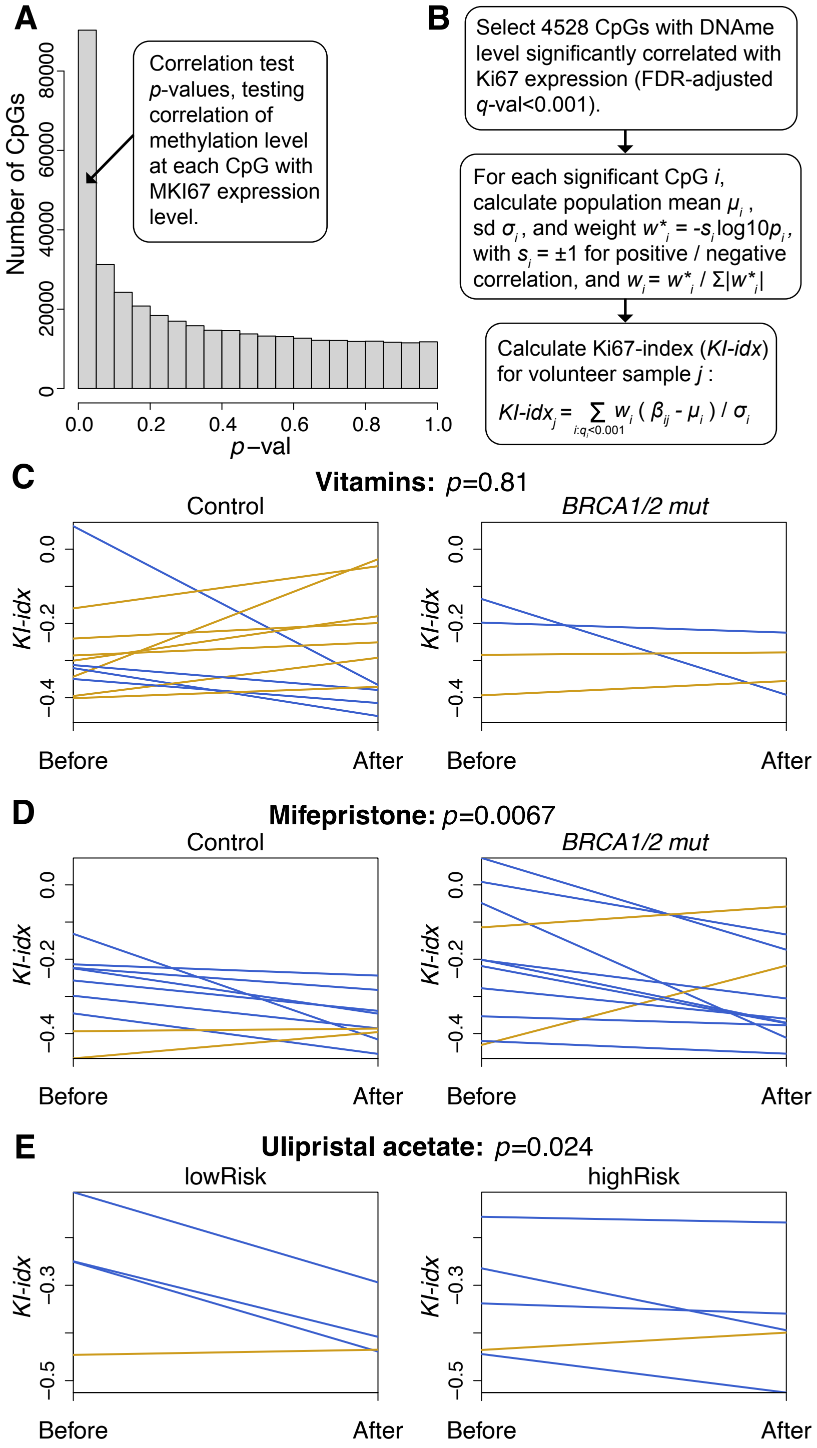

Fig. S9. The Ki67 index.

a A significant association between the methylation level of several CpGs and the expression level of MKI67 RNA in TCGA tumour samples was observed. **b** The Ki67-index (*KI-idx*) was derived from the most significant CpGs **c** There was no reduction in the *KI-idx* after administration of vitamins, however there was a significant reduction in the *KI-idx* after administering anti-progestins **d** mifepristone and **e** ulipristal acetate.

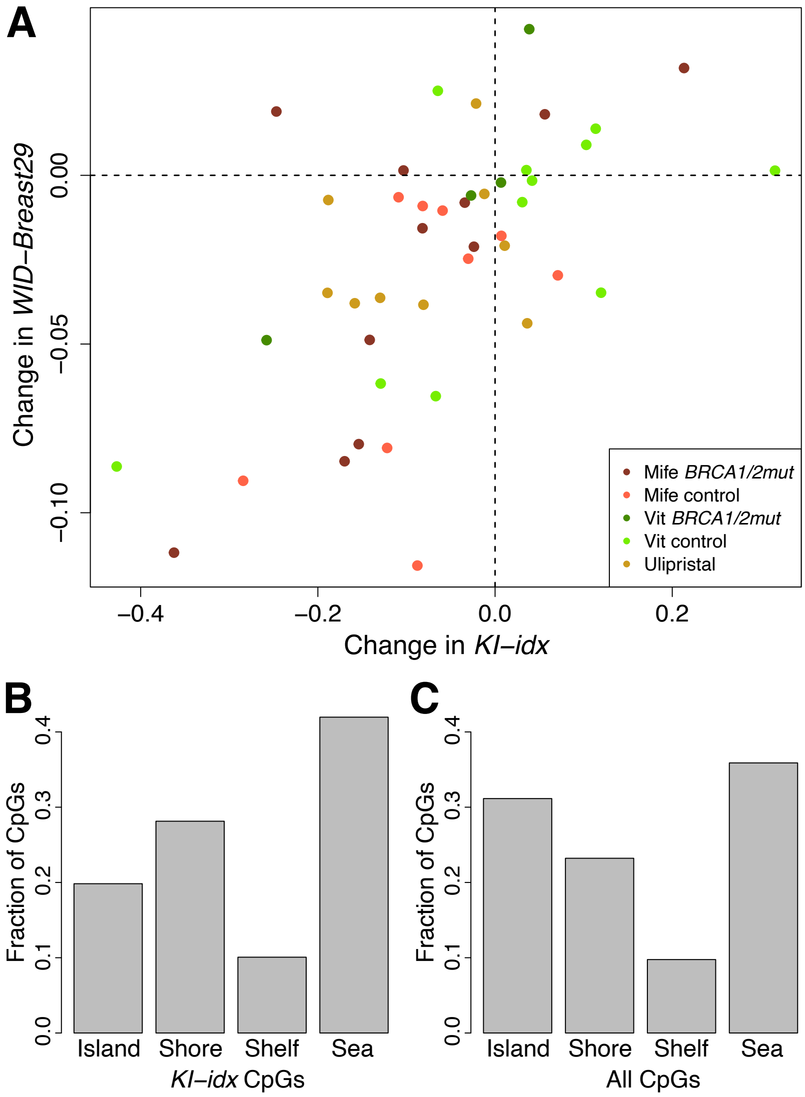

Fig. S10. Comparing the Ki67 index with the *WID-Breast29* index.

a Correlation of changes in the *KI-idx* with changes in *WID-Breast29*. **b** Proportion of the *KI-idx* CpGs with regards to location **c** proportion of all CpGs on the Illumina 450K array with regards to location.

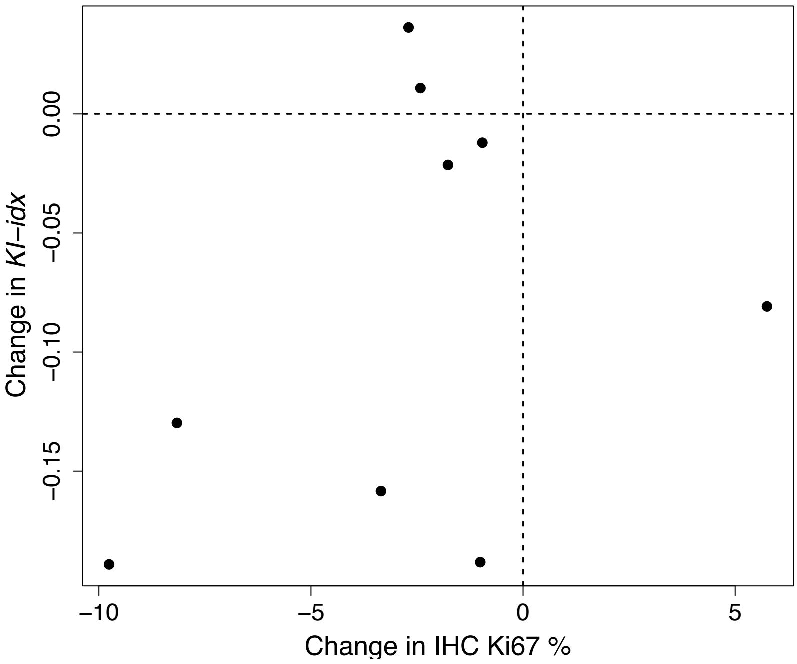

Fig. S11. Comparing the change (before and after ulipristal acetate) in percentage of immunohistochemistry Ki67-positive cell with change in Ki67-index in DNAme Dataset 4 (Clinical trial 3, '*A pilot prevention study of the effects of the anti-progestin ulipristal acetate (UA) on surrogate markers of breast cancer risk*').

SUPPLEMENTARY TABLES:

Table S1a. Summary statistics about the 20 healthy women (12 *BRCA1* mutation carriers and 8 *BRCA1* wild type) enrolled in the trial to measure estradiol and progesterone levels throughout the menstrual cycle (see also Fig.1 and 2).

BRCA1/2 status (statistic)	Cycle length / days	Age / years
BRCA1 mutant (min)	21	25
BRCA1 mutant (1st Qu.)	26.5	28
BRCA1 mutant (median)	27	35.5
BRCA1 mutant (mean)	27.7	33.9
BRCA1 mutant (3rd Qu.)	28.5	39.2
BRCA1 mutant (max)	34	42
BRCA1 wild type (min)	22	27
BRCA1 wild type (1st Qu.)	26	29.8
BRCA1 wild type (median)	28	32
BRCA1 wild type (mean)	27.6	34.5
BRCA1 wild type (3rd Qu.)	29	39.2
BRCA1 wild type (max)	33	45

Table S1b. Summary statistics about the 21 healthy BRCA mutation carriers enrolled in Clincal Trial 2 who provided samples for DNAme analysis as part of DNAme Set 3.

BRCA1/2 status (statistic)	Age / years
BRCA 1/2 mutant (min)	22
BRCA 1/2 mutant (1st Qu.)	30
BRCA 1/2 mutant (median)	34
BRCA 1/2 mutant (mean)	34.2
BRCA 1/2 mutant (3rd Qu.)	38
BRCA 1/2 mutant (max)	51

Table S2. The 37 *WID-Breast29* CpGs.

Δ median β BRCAmut - control	Illumina ID	Chromosome	BP locus	Gene symbol
0.079	cg07621749	15	41806141	*LTK*
0.067	cg09137696	16	56672415	*MT1A*
0.04	cg19283196	4	48485233	*SLC10A4*
0.036	cg03045635	4	9783198	*DRD5*
0.036	cg09578028	2	176987411	*HOXD9*
0.034	cg23847712	4	9783196	*DRD5*
0.034	cg14456683	3	147127010	*ZIC1*
0.03	cg25694447	3	157824150	*SHOX2*
0.028	cg03874199	2	176964456	*HOXD12*
0.022	cg15272362	20	25062823	*VSX1*
0.022	cg24154839	4	46995741	*GABRA4*
0.022	cg21269843	5	76934696	*OTP*
0.021	cg18438944	16	56672525	*MT1A*
0.021	cg01537995	14	85996352	*FLRT2*
0.018	cg26129769	3	157824148	*SHOX2*
0.017	cg06469345	4	9783246	*DRD5*
0.017	cg05093169	11	71955401	*PHOX2A*
0.016	cg01185626	1	75594016	*LHX8*
0.016	cg07557260	8	132052870	*ADCY8*
0.015	cg10343742	3	137483506	*SOX14*
0.014	cg25993718	20	54580196	*CBLN4*
0.014	cg17389504	10	28034853	*MKX*
0.013	cg03111498	20	25062860	*VSX1*
0.013	cg19711579	2	182545562	*NEUROD1*
0.013	cg01830294	7	116963492	*WNT2*
0.013	cg02631468	20	25062777	*VSX1*
0.012	cg22600043	8	109095991	*RSPO2*
0.012	cg14991487	2	176987404	*HOXD9*
0.012	cg17576288	13	79177877	*POU4F1*
0.012	cg05158197	4	168155888	*SPOCK3*
0.011	cg04690395	2	19558507	*OSR1*
0.011	cg08530317	11	20620824	*SLC6A5*
0.011	cg08961408	4	106816564	*NPNT*
0.01	cg16800165	14	85996364	*FLRT2*
0.01	cg00060320	3	134369974	*KY*
0.01	cg25116388	17	46692422	*HOXB8*
0.01	cg11723848	4	96470286	*UNC5C*

Table S3. Summary statistics about the donors of the breast cancer biopsies and matched surrounding normal tissue samples.

a: The 14 ER-ve/PR-ve/HER2-ve TNBC biopsies.

Age / years (min)	32
Age / years (1st Qu.)	38.8
Age / years (median)	44
Age / years (mean)	43.1
Age / years (3rd Qu.)	46.8
Age / years (max)	54

b The 31 ER+ve/PR+ve biopsies.

Age / years (min)	30
Age / years (1st Qu.)	46
Age / years (median)	51
Age / years (mean)	51.7
Age / years (3rd Qu.)	57
Age / years (max)	86

Table S4. The reference CpGs identified for luminal progenitor, mature luminal, and basal breast epithelial cells.

Lumin prog CpG	Lumin prog gene	Lumin mat CpG	Lumin mat gene	Basal CpG	Basal gene
cg04960880	GABBR1	cg00318877	METT5D1	cg00656991	FAM135A
cg07850274	RRH	cg02776119	MKLN1	cg01613725	
cg10716760	UBE2U	cg05686467	HEPN1	cg02389634	MIR2117
cg11259254	SGMS1	cg06058699	CD96	cg03074579	RCBTB1
cg12510203	GTDC1	cg06525193		cg06517794	USP33
cg14453449	ATP9B	cg06681656		cg08678429	CHD6
cg14968388	SIPA1L3	cg07703701	PDE4DIP	cg09433558	PLD1
cg16129213	SOD1	cg11180129	ALKBH1	cg11116035	HOMER1
cg17375269	RORA	cg13312976		cg12555369	USMG5
cg19517912	TIAM1	cg13983849	TBC1D19	cg13217184	PRH1;PRR4
cg19857361	CSMD2;HMGB4	cg13985135	YY1AP1	cg13713148	EGR3
cg20103263		cg15084470	SNRNP40	cg20626840	FAM82A1
cg23741159	TNPO3	cg16252122	PPHLN1	cg20834587	SH3RF1
cg24644113	TADA1	cg16377367	BCL10	cg21250061	ZBTB5
cg24828322	CYP2C8	cg18393905	RICTOR	cg21579982	FAF1
cg24894318		cg19241089	RBMS1	cg22172309	NEK1
cg25345805	IGF1R	cg19712745	EPB41L2	cg24326223	GOLGA4
cg26576172	ADAMTS3	cg19793807		cg24688636	SGK1
		cg20996887	SMYD3		
		cg22146642	MAD1L1		
		cg23501468	PSEN1		
		cg23934819	HEPN1		

Table S5. Summary statistics about the 15 healthy women who provided samples for RT-PCR and RNA-seq before and after mifepristone treatment.

BRCA1/2 status (statistic)	Cycle length / days	Age / years
BRCA 1/2 mutant (min)	25	21
BRCA 1/2 mutant (1st Qu.)	28	24
BRCA 1/2 mutant (median)	29	30.5
BRCA 1/2 mutant (mean)	28.9	30.4
BRCA 1/2 mutant (3rd Qu.)	30	36.2
BRCA 1/2 mutant (max)	31.5	41

REFERENCES

1. Papaikonomou K, Kopp Kallner H, Soderdahl F, Gemzell-Danielsson K: **Mifepristone treatment prior to insertion of a levonorgestrel releasing intrauterine system for improved bleeding control - a randomized controlled trial**. *Hum Reprod* 2018, **33**(11):2002-2009.
2. Teschendorff AE, Marabita F, Lechner M, Bartlett T, Tegner J, Gomez-Cabrero D, Beck S: **A beta-mixture quantile normalization method for correcting probe design bias in Illumina Infinium 450 k DNA methylation data**. *Bioinformatics* 2013, **29**(2):189-196.
3. Krueger F, Andrews SR: **Bismark: a flexible aligner and methylation caller for Bisulfite-Seq applications**. *Bioinformatics* 2011, **27**(11):1571-1572.
4. Yang Z, Wong A, Kuh D, Paul DS, Rakyan VK, Leslie RD, Zheng SC, Widschwendter M, Beck S, Teschendorff AE: **Correlation of an epigenetic mitotic clock with cancer risk**. *Genome Biol* 2016, **17**(1):205.
5. Teschendorff AE, Yang Z, Wong A, Pipinikas CP, Jiao Y, Jones A, Anjum S, Hardy R, Salvesen HB, Thirlwell C *et al*: **Correlation of Smoking-Associated DNA Methylation Changes in Buccal Cells With DNA Methylation Changes in Epithelial Cancer**. *JAMA Oncol* 2015, **1**(4):476-485.
6. Teschendorff AE, Breeze CE, Zheng SC, Beck S: **A comparison of reference-based algorithms for correcting cell-type heterogeneity in Epigenome-Wide Association Studies**. *BMC Bioinformatics* 2017, **18**(1):105.
7. Bartlett TE, Jia P, Chandna S, Roy S. **Inference of tissue relative proportions of the breast epithelial cell types luminal progenitor, basal, and luminal mature.** Scientific reports. 2021 Dec 8;11(1):1-0.
8. Zhu L, Lei J, Devlin B, Roeder K: **A Unified Statistical Framework for Single Cell and Bulk Rna Sequencing Data**. *Ann Appl Stat* 2018, **12**(1):609-632.
