# Supplementary material for: Antiprogestins reduce epigenetic field cancerization in breast tissue of young healthy women
Source: Genome Med. 2022 Jun 15;14:64. doi: 10.1186/s13073-022-01063-5 (PMC9199133; doi:10.1186/s13073-022-01063-5)
Supplement: Supplementary file 2 — Additional file 2. Is the protocol for Clinical Trial 1, ‘Mifepristone treatment prior to insertion of a levonorgestrel releasing intrauterine system for improved bleeding control – a randomized controlled trial’ (EudraCT registration number 2009-009014-40). [file 13073_2022_1063_MOESM2_ESM.pdf]

Trial Synopsis

Study Title	**Mifepristone treatment prior to insertion of a levonorgestrel releasing intrauterine system for improved bleeding control – a randomized controlled trial**
ClinicalTrial registration number<br><br>EuraCT number	EudraCT number 2009-009014-40.<br><br>Regional ethical review board at Karolinska Institutet permit 2009/144-31/4.
Clinical study phase	Phase IV
Study objective	To study the effect of pre-treatment with mifepristone on the initial bleeding pattern in women during three months after insertion of the LNG-IUS.<br>Secondary objectives include the effect of mifepristone on the breast tissue and effects of mifepristone and LNG on the endometrium. An endometrial biopsy will be obtained at baseline prior to start of pretreatment and at three months following placement of the LNG-IUS 52mg.<br>After inclusion the women will be randomized to a 2 month treatment with mifepristone or an comparator with no effect on the bleeding pattern or endometrium (i.e B-vitamin tablets). Endometrial and breast biopsies will be obtained in the luteal phase prior to the start of treatment. The breast biopsy will then be repeated after mifepristone treatment while the second endometrial biopsy will be carried out after 3 months with LNG-IUS52mg treatment. Besides a vaginal ultrasound scan in the beginning of the study an ultrasound scan will be performed prior to the endometrial biopsies.<br>A core needle breast biopsy is collected at baseline and following two months of pretreatment prior to IUS placement.
Intervention arm	Pre-treatment with a low dose mifepristone (50 mg every second day) for two months prior to insertion of a LNG-IUS 52 mg used as a contraceptive method.
Reference arm	Inactive comparator (TrioB)
Study Design	A prospective, randomized two armed parallel open label trial.

Main patient inclusion criteria	1. Women 18 years of age or above eligible for LNG-IUS<br>2. Regular and normal menstrual cycles lasting 25–35 days,<br>3. Willing and able to participate after the study has been explained<br>4. Signed informed consent
Main patient exclusion criteria	1. Allergy or contraindication to mifepristone or LNG-IUS<br>2. A medical condition or disease that requires special treatment, care or precaution (e.g. corticosteroid or anticoagulant therapy)
Number of patients	58 women
Primary variable	Days of bleeding and spotting in both groups
Statistical analysis	Patient flow during trial will follow the CONSORT chart. For demographic variables the full analysis set, FAS (all subjects randomized), will be presented with descriptive statistics. All subjects randomized having known primary outcome will be included in the main analysis in their randomized groups. If attempts fail to locate and contact a woman lost to follow-up, and her final outcome of treatment is not known that woman will be excluded from the main analysis.<br><br>Crude rates for each outcome will be calculated in each arm and exact confidence intervals calculated.
Study duration	Study initiation November 2009 with the last subject screened November 2013 and last study contact January 2015.
