# Supplementary material for: Antiprogestins reduce epigenetic field cancerization in breast tissue of young healthy women
Source: Genome Med. 2022 Jun 15;14:64. doi: 10.1186/s13073-022-01063-5 (PMC9199133; doi:10.1186/s13073-022-01063-5)
Supplement: Supplementary file 3 — Additional file 3. Is the protocol for Clinical Trial 2, ‘The effect of a progesterone receptor modulator on breast tissue in women with BRCA1 and 2 mutations’ (EudraCT registration number: 2012-003703-35). [file 13073_2022_1063_MOESM3_ESM.pdf]

CONFIDENTIAL

Trial title: **The effect of a progesterone receptor modulator on breast tissue in women with BRCA-1 and -2 mutations - a placebo controlled RCT.**

Short title: **BRCA1/2 and effect of mifepristone on the breast**

Clinical Trial Id Number:
EudraCT Number: 2012-003703-35

- **Trial sponsor**
  Kristina Gemzell-Danielsson.

  Sponsor's Protocol Code Number: **W2012B (prior WMB12)**

- **Principal investigator**

  Kristina Gemzell-Danielsson, MD Ph D
  Department of Woman and Child Health,
  Division of Obstetrics and Gynaecology,
  Karolinska Institutet,
  Karolinska University Hospital,
  SE-17176 Stockholm, Sweden
  telephone: + 46 8517 72128
  telefax no.: +46 8517-74314
  email: kristina.gemzell@ki.se

PARTICIPATING CENTRES: 1

Principal investigator's signature:

Date: August 10, 2012

The effect of a progesterone receptor modulator on breast tissue in women with BRCA-1 and -2 mutations - a placebo controlled RCT.

Purpose and Aims

Ovarian steroids, as well as their synthetic counterparts gestagens and estrogens have a role in breast cell proliferation and the development of breast cancer. Here, the effect of a progesterone receptor modulator, mifepristone, on cell proliferation in human breast tissue in vivo will be studied in women with BRCA-1 or -2 mutations. Our preliminary results implicate a possible protective effect of mifepristone in breast epithelium. The ability of mifepristone to block breast epithelial cell proliferation may prevent tumorigenesis and may also prove beneficial when used for contraceptive purposes and on other indications. The proposed project concerns a RCT on mifepristone vs. placebo treatment of women with BRCA-1or -2 mutations with a high risk/incidence of breast cancer and ovarian cancer

---

Survey of the field

Women with mutations in the breast cancer susceptibility genes BRCA-1 and -2 are predisposed to breast and ovarian cancers. The breast is a target organ for estrogens and progestins, but the multifactorial mechanisms which regulate breast cell proliferation and apoptosis are still incompletely understood. In contrast to the effect in the endometrium, adding progestins to estrogen therapy does not seem to have any antiproliferative, protective effect in the breast (1). In the endometrium, estrogens increase cell proliferation and this effect is antagonized by progestins. In post-menopausal women, it is well established that the risk of hyperproliferation and endometrial cancer following unopposed estrogen can be counteracted by progestins (2). However, in breast tissue this is not the case; conversely in breast tissue / epithelium progestins act as mitogens (3, 4). Moreover, in hormone therapy (HT) of postmenopausal symptoms, the progestin component of the HT has emerged as a significant risk factor for breast cancer. Even relatively short-term combined estrogen plus progestin use increases the incidence of breast cancer, which is diagnosed at a more advanced stage, and increases the percentage of women with abnormal mammograms. Thus results suggest that estrogen plus progestin stimulate breast cancer growth and hinder breast cancer diagnosis significantly more than estrogen alone (5). A more than 4-fold increase in the percentage of Ki-67 positive cells, a marker of cell proliferation, has been demonstrated in breast biopsies already at 3 months of continuous combined estrogen/progestin therapy in post-menopausal women (6).

In premenopausal women, the effect of combined hormonal contraception on the breast may increase the risk of breast cancer (7). A 2-fold increase in the Ki-67 index was observed in women using combined oral contraception compared with non-users. Furthermore, it was noticed that some pill users reacted with an extreme elevation of Ki-67 index up to 50% (8). It has been indicated that women with BRCA-1 or -2 mutations are at a higher risk for breast cancer if exposed to combined contraceptive pills (9).

Although the knowledge on the effects of estrogens in the breast is limited, the effects of gestagens is even less known and studied. Epidemiological studies show an increased risk for breast cancer in post menopausal women, who use hormone replacement therapy after longtime use (more than five years). Recent studies indicate that the gestagen component might be more important than the estrogen component for the risk of developing breast cancer (10, 11).

Mifepristone is a synthetic 19-norsteroid that binds with high affinity to the progesterone receptor thereby preventing the effect of progesterone. The effects of progesterone receptor modulators (PRMs), such as mifeprsitone, during the menstrual cycle depend on the timing of treatment and the dose used. PRMs have been shown to act as a possible estrogen free contraceptive method (12) through its antiovulatory effect as well as an effect on the endometrium. Furthermore, PRMs can improve bleeding patterns in women using gestagen only contraceptives (13). On the basis of the observed effects in the endometrium, there is also an increasing interest in the clinical application of PRMs in post-menopausal hormone therapy (14, 15). However, despite a number of studies on the effect of PRMs in the endometrium, there is limited knowledge on the effect on the breast tissue in fertile women *in vivo*.

Recent advances in the field of molecular genetics have provided a molecular basis for the concept that cell division is essential in the complex process of the genesis of human cancer. Cell division per se increases the risk of genetic errors of various kinds. The activation of oncogenes, whether by mutation, translocation or amplification, requires cell division (16). In an *in vitro* breast cancer cell line, PRM exposure resulted in an apoptotic effect through growth arrest and Caspase activation (17).

BRCA 1/2 mutations and the risk of breast cancer

Women with mutations in the breast cancer susceptibility genes BRCA-1 and -2 are predisposed to breast and ovarian cancers. Progesterone receptors are over expressed in the mutant mammary epithelial cells because of a defect in their degradation by the proteasome pathway. Mutations in the BRCA -1 and -2 tumor suppressor genes are responsible for approximately 3-5% of all breast cancer. However, the lifetime estimates of breast cancer risk among these women range from 36 to 90% (18)). The individual risk may vary substantially according to various background factors such as presence or absence of history of breast cancer. Breast tissue collected from prophylactic mastectomy specimens also display an increased prevalence of premalignant or malignant lesions suggesting presence of a preinvasive phase of the BRCA-1 or -2 mutation associated breast cancer (19, 20)).

Various risk reducing strategies, such as annual screening, prophylactic mastectomy and/or salpingo-oophorectomy and chemoprevention has been used to lower the risk of breast cancer among carriers of BRCA-1 or -2 mutations. A recent prospective study reported no breast cancers among 247 women with risk-reducing mastectomy whereas 98 cases of breast cancer were seen in 1372 BRCA1/s carriers who did not undergo mastectomy (21).

In the animal model of BRCA-1 mutation, the BRCA1/p53-deficient mice, treatment with mifepristone prevented mammary tumerogenesis (22). Recently we have shown an antiproliferative effect of low dose mifepristone in human breast tissue *in vivo* (23). Thus, a protective effect of PRMs in breast tissue, could be hypothesized. This might also provide an additional low risk and patient friendly risk-reduction strategy among women with high risk of breast cancer.

Objectives

- **Research objective**

  To study the safety and effect of treatment with mifepristone, a progesterone receptor modulator, on epithelial cell proliferation in human breast tissue in women with BRCA-1 or -2 mutations prior to protective mastectomy.

Project description

- **Hypothesis/ Theory**

  Mifepristone treatment exerts an antiproliferative, protective effect on breast tissue in women with BRCA-1 or -2 mutations

Study Design

Randomized, double blind, placebo controlled trial. Women will be recruited among patients with BRCA-1 or -2 mutations scheduled for prophylactic mastectomy. Included women will be randomized to a 3-month treatment with mifepristone, 50 mg (Mifegyne, Exelgyn, Paris, France) or placebo taken orally every second day. Breast biopsies will be obtained in the luteal phase prior to start of treatment and again during surgery. Women who are not sterilized or whose partner is not sterilized, and who are not using and IUD will be instructed to use barrier methods for contraception during the treatment period. Hematology, safety lab, hormonal values and endometrial histology (in biopsies) will be analyzed at the time of breast biopsies. Women will keep daily records of bleeding pattern and side effects and will return for follow-up visits according to the routine screening for these patients. Women will be able to serve as their own controls in addition to the two study groups.

Study outcomes

<u>Main outcome:</u>

Effects of mifepristone on epithelial cell proliferation in breast tissue of women with BRCA1 - and/or 2 mutations

<u>Secondary outcomes:</u>

- Vital signs and safety lab analysis
- Side effects and Adverse Events
- Endometrial effects (bleeding patterns, endometrial morphology)
- Ovarian effects

- Acceptability
- Breast symptom evaluation (Breast symptom score (23))
- Expression of estrogen receptor (ER), progesterone receptor (PR), androgen receptor (AR) as well as apoptosis, proliferation and biomarkers for the development of cancer in the breast tissue and collagen content, before and at the end of three months mifepristone or placebo treatment.

Study population

Inclusion criteria:

- Pre-menopausal women, >/= 18 years of age
- with good general health and
- regular menstrual cycles (25-35 days) who are willing and
- able to participate after giving informed consent.
- women having BRCA1/2 mutation and have decided to undergo risk reducing mastectomy

Exclusion criteria includes:

- Any hormonal treatment used within 2 months prior to study start and
- Any contraindication to mifepristone

Primary end point: 20% reduction in breast cell proliferation at 12 weeks treatment
Secondary endpoints:

- Type of Side effects and Adverse Events
- Endometrial morphology and rate of PEAC (progesterone receptor modulator associated changes)

Sample size calculation: Power calculation is based on our earlier results in premenopausal women. 20 women per group will be enough to show a significant reduction in breast proliferation during mifepristone treatment (23). The study is a proof-of- concept study. Due to the relatively low rate of BRCA mutations and the fact that women can serve as their own controls, randomization will be done in a 2:1 fashion. To compensate for loss to follow up and inadequate biospy material 30 women will be included in the BRCA group while 15 women will be randomized to TrioB treatment.

Description of the study drug
The low-dose regimen of mifepristone (50 mg every second day) is the same as studied for the treatment of uterine leiomyoma and effect in premenopausal human breast tissue (24)

Mifepristone

a) Chemical name: 17beta-hydroxy-11beta-[p-(dimethylamino)-phenyl]-17-(1-propynyl)estra-4,9-dien-3-one

Empirical formula: $C_{29}H_{35}N_1O_2$

b) Route of administration: oral tablets
c) Amount present per tablet: 200 mg (divided into 4 parts of approximately 50mg)

Comparator: visually identical TrioB tablets (Recip, Stockholm, Sweden) (a quarter of a tablet every second day).

Tablets will be purchased, labeled and distributed via the University Hospital Pharmacy. The rationale for the mifepristone regimen is that this dose has been shown to be effective in reducing breast cell proliferation in premenopausal women and safe when used for the treatment of uterine leiomyoma with good tolerability during 3 months treatment.

- **Subject allocation and randomization**

Randomisation will be done in a 2:1 fashion. At screening women will undergo general examination including blood pressure, height and weight, haematology, kidney-, liver function tests, hormonal values (FSH, LH, PRL) and thyroid function. A gynecological examination including chlamydia test, pap smear and vaginal ultrasonography with measurements of endometrial thickness. An endometrial biopsy will be obtained. A mammography will be performed and an ultrasound guided core needle breast biopsy taken.

Women will be randomized to treatment with 50 mg mifepristone every second day or TrioB. Women randomized to mifepristone (Mifegyne, Exelgyn, Paris, France) (n=30) or placebo (n=15) will be given a quarter of a tablet, every second day of for 3 months. Tablets will be handed out in a special box, for one week at a time, and women will be instructed to come back to the centre to receive new tablets every week during the treatment period. Compliance will be checked every week when the women come to get new tablets. Empty boxes or blisters and any remaining tablets will be brought back by the women to the site at each visit.

The women will report on the treatment effects on breast symptoms once weekly and any side effects and AE/SAE during three months´ treatment. During the last week of treatment, a general and gynecological examination will be performed as before the study. During surgery an endometrial biopsy will be obtained. Endometrial evaluation will be done by Prof Alistair Williams, a pathologist who is an international expert on PRMs and the endometrial effects. Breast tissue will undergo pathological evaluation according to the clinical routine. Tissue will be provided by the pathologists for the proposed analyses.

- **Planned laboratory analyses**

1. Differential expression of genes specifically in the pathways involving apoptotic and cell proliferation will be studied with microarray. Microarray data will be analyzed using bioinformatics software Ingenuity pathway analysis (IPA) available with the

group to identify the major canonical pathways altered with the treatment. In-depth data mining will be performed to fish out the genes belonging to cell proliferation, cancer and cell death pathways.

2. Significantly altered factors observed in the microarray study, including PTEN, Bcl-2 and Ki-67 along with steroid receptors (ERα, ERβ, ERβcx, PR-A, PR-B, AR), as well as collagen content will be reconfirmed by real time PCR and the protein expression studied by immunohistochemistry using breast tissues collected at the beginning and end of treatment.

Breast tissue collected with the core needle biopsy and at the time of surgery will be snap frozen for gene expression studies and a small portion of breast and endometrial tissue will be fixed in 10% formalin for immunohistochemical analysis. Total RNA will be extracted using Trizol method (we have standardized it for breast tissues) and microarray will be performed at core facility using Human Gene 1.1 ST array (Affymetrix). The data will be filtered using PCA and analyzed using IPA. Immunohistochemical analysis will be done using standardized protocol in the lab (23)

.

- **Safety data**

Safety data includes vital signs, general- and gynecological-, incl breast - examinations, safety lab (hematology (blood status+CRP), kidney function (Na, K, krea) liver function (ASAT, ALAT, ALP, GT, bilirubin) , thyroid function (TSH,T3,T4), hormonal values FSH ,LH , PRL, SHBG, testosterone, E2 (sensitive) progesterone, urine dipstick and pregnancy test (prior to start). Endometrial histology will be investigated in biopsies obtained at baseline and at surgery. Women with BRCA-1/-2 mutations are followed according to the existing clinical routine with regard to ovarian cancer screening.

- **Side effects**

Side effects of mifepristone are mild and the only significant side effect reported in previous clinical trials with the same regimen has been mild flushes. All side effects and SAE/AE as well as any concomitant medication will be recoded by the participating patients in a dairy.

- **Materials and Project management**

The trial will be conducted under the supervision of the principal investigator who are also responsible for the analysis and writing-up of the results in collaboration with the co-investigators. One research nurse not directly involved in the trial will be responsible for monitoring the study on a monthly basis.

Data management and analyses. Descriptive statistics will be calculated for all baseline characteristics for all subjects included in the main analysis, by treatment group, to assess comparability of the groups. The overall summary statistics will be compared with those for the subjects lost to follow-up. The study is a hypothesis generating study.

Biological material will be stored in bio banks until analysed. Permission will be obtained from participants for sending material for analyses abroad as specified in the informed consent.

Data from hospital records and other data generated from the study will be entered into CRFs (case record files) stored at the research centre.

Time plan:
It is estimated that ethics permission and permission form the medical products agency will be granted in Q3-4, 2012. recruitment of patients will start jan 2013 and is estimated to be concluded within 2 years. Time between the last patient recruited and the last patient last visit (LPLV) will be between 3 to 4 months. Lab- and data -analysis and presenting will take another one year. The experimental and hypothesis generating studies will form the basis for designing further clinical trials.

Implementation:
The project will be carried out as a RCT conducted at the WHO-centre, Dept. of Women's and Children's Health, division of Obstetrics and Gynecology Karolinska Institutet and Karolinska University Hospital, Stockholm, Sweden. The research team has extensive experience with clinical trials and studies on PRMs. There is a well established team of gynecologists, oncologists and surgeons for care of women with BRACA -1- and -2 mutations.

The annual number of women with BRCA1,-2 mutations undergoing prophylactic mastectomi is approximately 20. Breast biospies will be obtained and analysed within an existing research collaboration and network at KI and in Houston, Texas.. Prof. Jan-Åke Gustavsson is world leading in steroid hormone receptor research with excellent facilities for the planned receptor analysis as Director, Center for Nuclear Receptors and Cell Signalling, Department of Biology and Biochemistry - University of Houston (80%) and Novum, Karolinska Institutet (KI) (20%). The group has established collaboration with proteomic- and mutation analysis core facilities at KI. The research team has long experience in handling human breast samples and research on hormonal influence on the human breast. The clinic has a well established specialized team caring for women with BRCA-1 and -2 mutations

Role of the co-workers in the project:
Kristina Gemzell Danielsson is professor of Obstetrics and Gynecology and Director of the WHO centre for Human Reproduction with expertise in endometrial function, fertility/infertility and contraceptive development. She is an international recognized expert within Human Reproduction and a member of major international organizations in the field incl. WHO and ICCR (International committee for contraceptive research) Population Council/ Biomedical program, Rockefeller University, NY, USA. She is Head of FRH lab which houses expertise in reproductive physiology/ endocrinology and all laboratory methods necessary for the project. In the research group there are senior researchers, post docs, PhD students, two research midwifes and four laboratory technicians.

Angelique Flöter Rådestad,MD Senior consultant in obstetrics and gynecology and subspecialist in gynecological tumour surgery. She has a broad clinical experience and responsability for the patient population that will be included in the study. She has clinical

and scientific expertise in endocrinology, hormonal replacement and quality of life after prophylactic operations. Several of her ongoing research projects in gynecological tumours are translational. She will be recruiting, councelling and performing the clinical work during the study.

PGL Lalitkumar, hold a PhD in reproductive physiology. He is senior researcher and embryologist responsible for the FRH-lab. He is supervising the experimental work.

Our collaborators
Associate Professor **Edvard Azavedo,** , senior consultant, expert in breast pathology, ultrasound, mammography and breast biopsy technique.
Dr. Inkeri Leonardsson Schultz, MD, consultant in reconstructive surgery is repsonsible for mastectomy/breast reconstructions.
Professor J-Å Gustavsson, Karolinska Institutet, is world leading in research on steroid receptors. He is head of a large research group a KI and in Houston, Texas, which attracts extended international research collaborations and exchange.
Professor Alistair Williams, Pathologist, Edinburgh University, UK is a collaborator and world leading expert on endometrial effects of progesterone receptor modulators.

Ethical issues
The RCT will be posted at **www.glinicaltrials.gov.** The study will be conducted according to GCP and the Helsinki declaration.

Women will receive oral and written study specific information and an informed consent will be signed by the participating women and the investigators prior to any participation in the trial. PUL (personuppgiftslagen) will be followed and bio bank information provided. All biological material collected related to the study will be stored in bio banks. Confidentiality will be guaranteed. Data recorded on CRFs will be coded and stored with the PI in approved and locked storages. Data will only be presented anonymous and at group levels.

*Risk benefit analysis: Participation will be v*oluntary and subjects have the right to withdraw from the study at any time without prejudice to their further medical care.
Women with BRCA-1 mutations also face a higher risk of ovarian cancer. The antiovulatory effect of mifepristone offers a possible benefit in prevention. Women will be followed according to the clinical routine with frequent assessments.
There is no previously described AE connected to the proposed procedures other than possible hematoma after venous puncture and breast biopsy. Previous studies have shown that treatment with 50 mg mifepristone every second day for a period of 3 months is safe.

AE/SAE

The treatment will be discontinued if severe side effects or symptoms develop that, in the opinion of the investigators, constitute a threat to the woman's health. The reason for discontinuation will be recorded in the clinical dossier. Experienced side effects and any

medication or treatment will be reported by the patients in a diary on a weekly basis. Any unexpected AE (with the study drug) and SAE will be reported electronically to the Eudra-Vigilance-database. This will also be recorded in the patient file which will be kept (with the other study documents) for at least 10 years.

Significance

The study will confirm the safety of mifepristone in women with BRCA-1 and -2 mutations. The PRM, mifepristone, has recently been shown to have an antiproliferative effect in breast tissue in premenopausal women. So far there is no data in women with an increased risk of breast cancer associated with BRCA-1 and -2 mutations. An antiproliferative effect of PRMs could potentially be used to develop hormonal treatment options that would offer protection of the breast which would allow avoidance of prophylactic mastectomy. Since breast cancer is the most common cancer among women this may have significant clinical implications also if used for contraception, postmenopausal hormone treatment or on other indications. This would be of benefit for society by reducing costs from disease but also from repeated controls and prophylactic mastectomy but even more for the individual woman.

Clinical application/relevance.

Since breast cancer is the most common cancer among women this project may have significant clinical implications if shown to prevent cell proliferation and tumorgenesis in women with BRCA1 and 2 mutations. This would be of benefit for society by reducing costs from disease but also from repeated controls and prophylactic mastectomy but even more for the individual woman. The present study will form a bases for further clinical trials including women who chose not to undergo prophylactic mastectomy.

Key References

1. Santen RJ, Pinkerton J, McCartney C, Petroni . J Clin Endocrinol Metab 2001;86:16–23.
2. Weiderpass E, et al., JNatlCancer Inst 1999;91:1131–1137
3. Williams G, et al., Int J Cancer, 1991; 48: 206-10,
4. Hofseth L, et al., J Clin Endocrinol Metab, 1999; 84: 4559-65.
5. Chlebowski RT, et al. the WHI Randomized Trial. JAMA 2003;289:3243–3253.
6. Conner P, et al., Breast Cancer Res Treat 2003;78:159–165.
7. Collaborative Group on Hormonal Factors in Breast Cancer. Lancet 1996;347:1713–1727.
8. Isaksson E, et al., Breast Cancer Res Treat 2001;65:163–169.
9. Olsson H eta l., Hum Mol Genet. 2011 Aug 15;20(16):3304-21
10. Hillman JJ, et al J Womens Health 2004, 13(9):986-92
11. Chen CL, et al Jama 2002, 287:734-41
12. Gemzell-Danielsson K et al., Hum Reprod 1993;8:870–873.
13. Gemzell-Danielsson K, et al., Hum Reprod 2002;17:2588–2593.
14. Cameron ST, et al.,. Steroids 2003;68:1053–1059.
15. Slayden OD, et al., Hum Reprod 2006;21:3081–3090.
16. Preston-Martin et al., Cancer Res 1990;50:7415–7421.
17. Gaddy VT, et al., Clin Cancer Res 2004;10:5215–5225.

18. Tung N. JAMA 2011; 305: 2211-20
19. Isern AE et al. Eur J Surg Oncol 2008; 34: 1148-54
20. Kauff ND et al., Cancer 2003; 97: 1601-8
21. Domchek et al., JAMA 2010; 304: 967-75
22. Poole et al., Science 2006
23. Engman M, et al., Hum Reprod 2008;2:2072-9.
24. Engman M, et al., Hum Reprod 2009; Apr 23
